# Neoadjuvant versus perioperative immunotherapy in resectable NSCLC: A PD-L1–stratified Bayesian network meta-analysis

**DOI:** 10.3389/fonc.2026.1905730

**Published:** 2026-08-06

**Authors:** Yi Jian, Zhijie Wu, Feihong Lai, Moluyu Wei, Jin Cao, Panhong Jia, Mei Li, Cao Qing, Fashuang Yang, Ke Wang

**Affiliations:** 1 Department of Pulmonary and Critical Care Medicine, The First Affiliated Hospital of Guangxi Medical University, Nanning, Guangxi, China; 2 Department of Geriatrics, Jinsha County People’s Hospital, Bijie, Guizhou, China

**Keywords:** Bayesian network meta-analysis, neoadjuvant chemoimmunotherapy, non–small cell lung cancer, PD-L1, perioperative chemoimmunotherapy

## Abstract

**Background:**

Neoadjuvant immunochemotherapy (NIC) and perioperative immunochemotherapy (PIC) improve outcomes in resectable non-small cell lung cancer (NSCLC), but their relative effects across programmed death-ligand 1 (PD-L1) subgroups remain unclear because head-to-head trials are lacking. This study aimed to compare NIC, PIC, and platinum-based chemotherapy (CT) for event-free survival (EFS) and overall survival (OS), and to compare individual preoperative regimens for pathologic complete response (pCR) and major pathologic response (MPR), across PD-L1 subgroups.

**Methods:**

PubMed, Embase, the Cochrane Library, and Web of Science were searched through 17 January 2026 for randomized trials. Bayesian random-effects network meta-analyses estimated hazard ratios (HRs) for EFS and OS and risk ratios (RRs) for pCR and MPR, with 95% credible intervals (CrIs). Analyses were conducted in R (rjags and gemtc); funnel plots were generated in Stata 17.0.

**Results:**

Eight trials (10 publications; 3,608 patients) were included. Six publications were judged to have a low overall risk of bias, whereas four were judged to have some concerns. Among patients with PD-L1 <1%, PIC improved EFS versus CT (HR 0.74, 95% CrI 0.58–0.96), but neither PIC nor NIC improved OS (PIC: HR 0.93, 95% CrI 0.65–1.33; NIC: HR 1.32, 95% CrI 0.67–2.58). Among those with PD-L1 ≥1%, both PIC and NIC improved EFS (PIC: HR 0.51, 95% CrI 0.36–0.72; NIC: HR 0.44, 95% CrI 0.26–0.74) and OS (PIC: HR 0.57, 95% CrI 0.41–0.78; NIC: HR 0.47, 95% CrI 0.25–0.88) versus CT. In PD-L1 ≥50%, NIC was associated with longer EFS versus CT (HR 0.24, 95% CrI 0.06–0.87). In PD-L1 ≥1%, tislelizumab plus CT was associated with higher pCR versus CT (RR 11.07, 95% CrI 5.35–27.31), and camrelizumab plus CT was associated with higher MPR (RR 8.28, 95% CrI 3.58–21.94).

**Conclusion:**

Comparative estimates for neoadjuvant and perioperative immunochemotherapy differed by PD-L1 subgroup and outcome. Indirect comparisons and limited certainty preclude firm conclusions about the superiority of either strategy.

**Systematic review registration:**

https://www.crd.york.ac.uk/PROSPERO/, identifier CRD420261303366.

## 1 Introduction

Lung cancer remains a leading cause of cancer incidence and mortality worldwide, and non-small cell lung cancer (NSCLC) accounts for approximately 80%–85% of cases (1–5). Postoperative recurrence and distant metastasis remain common barriers to long-term survival (6–8). Platinum-based perioperative chemotherapy provides only a modest survival benefit, supporting the evaluation of more effective systemic strategies to eradicate micrometastatic and residual disease (9–15).

Randomized trials have established immunotherapy-based treatment as an important component of care for resectable NSCLC. CheckMate 816 demonstrated improved event-free survival (EFS) and pathologic complete response (pCR) with neoadjuvant nivolumab plus chemotherapy, whereas KEYNOTE-671, AEGEAN, CheckMate 77T, Neotorch, and RATIONALE-315 evaluated perioperative strategies that continued immunotherapy after surgery (9–11, 16–22). These trials primarily compared an immunotherapy-based strategy with chemotherapy rather than directly comparing neoadjuvant-only and perioperative approaches.

The central uncertainty is whether the relative effects of neoadjuvant and perioperative immunotherapy-based strategies differ across programmed death-ligand 1 (PD-L1) subgroups and endpoints. Tumor-cell PD-L1 may reflect adaptive immune suppression, but its predictive value is limited by spatial and temporal heterogeneity and by responses observed in PD-L1-negative tumors (9, 23–26). Pathologic outcomes are determined before postoperative therapy, whereas EFS and overall survival (OS) may reflect the entire perioperative strategy. The only CTLA-4-containing regimen in the eligible evidence was nivolumab plus ipilimumab, not CTLA-4 monotherapy. This combination retains direct PD-1 blockade, and CheckMate 816 reported outcomes within prespecified PD-L1 strata; PD-L1 was therefore treated as an effect modifier of a PD-1-containing regimen rather than as a direct biomarker of CTLA-4 inhibition (16, 27, 28).

This Bayesian network meta-analysis compared neoadjuvant and perioperative immunotherapy-based strategies with chemotherapy for EFS and OS across trial-defined PD-L1 subgroups and compared specific preoperative regimens for pCR and major pathologic response (MPR). The objective was to characterize subgroup-level comparative evidence rather than to identify an optimal treatment for individual patients.

## 2 Materials and methods

This network meta-analysis was conducted in accordance with the PRISMA extension for network meta-analyses (**Supplementary Table 1**) (29, 30). The absence of head-to-head RCTs directly comparing neoadjuvant-only and perioperative immunotherapy-based strategies justified the use of a Bayesian framework to synthesize indirect evidence and estimate treatment-ranking probabilities (31). The protocol was prospectively registered in PROSPERO (CRD420261303366) to support transparency and methodological rigor.

### 2.1 Data sources and search strategy

PubMed, Embase, the Cochrane Library, and Web of Science were systematically searched from database inception through January 17, 2026, without language restrictions. The search strategy combined controlled vocabulary with free-text terms, including “Carcinoma, Non-Small-Cell Lung,” “non-small cell lung cancer,” “Neoadjuvant Therapy,” “Chemotherapy, Adjuvant,” “PD-L1 inhibitor,” “PD-1 inhibitor,” “Immune Checkpoint Inhibitors,” and “randomized clinical trial” (**Supplementary Table 2**).

### 2.2 Eligibility criteria

Eligible studies were randomized controlled trials enrolling patients with histologically or cytologically confirmed resectable NSCLC and comparing a neoadjuvant or perioperative regimen containing a PD-1 or PD-L1 inhibitor, with or without chemotherapy, against conventional platinum-based chemotherapy. Studies were required to report EFS, OS, pCR, or MPR within at least one prespecified PD-L1 subgroup (<1%, ≥1%, 1–49%, or ≥50%). EFS and OS were evaluated at the strategy level, whereas pCR and MPR were evaluated according to the specific preoperative regimen.

A CTLA-4 inhibitor was eligible only as part of a PD-1-containing combination when PD-L1-stratified outcomes were available; CTLA-4 monotherapy was excluded. Nivolumab plus ipilimumab was classified with the neoadjuvant strategy for EFS and OS because both agents were administered exclusively before surgery, but it was retained as a distinct regimen for pCR and MPR. Nonrandomized studies, adjuvant-only immunotherapy studies, reports with unusable outcome definitions or data, and duplicate reports without additional relevant follow-up were excluded. For multiple reports of the same trial, the most complete dataset for each outcome was used without double-counting participants.

### 2.3 Data extraction

Three investigators independently extracted study and outcome data, and disagreements were resolved through discussion with a fourth investigator. Extracted variables included publication year, trial identifier, design, disease stage, histology, sample size, age, sex, race or ethnicity, PD-L1 assay and subgroup, treatment regimens, and reported outcomes. Published HRs and 95% confidence intervals for EFS and OS were converted to log HRs and standard errors. Arm-level event counts and sample sizes were extracted for pCR and MPR; an outcome network was analyzed only when the required subgroup data were available.

### 2.4 Risk of bias assessment

Risk of bias was assessed with the Cochrane Risk of Bias 2 tool across five domains: the randomization process, deviations from intended interventions, missing outcome data, outcome measurement, and selection of the reported result (32). Each domain and the overall judgment were classified as low risk, some concerns, or high risk. For open-label trials, outcome-measurement judgments were based on the RoB 2 signaling questions and trial-specific reporting; blinding of outcome assessors was considered only when it was explicitly reported.

### 2.5 Statistical analysis

Separate Bayesian random-effects consistency models were fitted for each outcome and PD-L1 subgroup in R using the rjags and gemtc packages (33). Reported log HRs and corresponding standard errors for EFS and OS were modeled using a normal likelihood with an identity link, whereas arm-level event counts for pCR and MPR were modeled using a binomial likelihood with a log link to estimate risk ratios (RRs) (34). In the absence of sufficiently robust external information to support informative priors, default vague priors implemented in gemtc were used to minimize undue prior influence on the posterior estimates. Basic relative treatment effects were assigned normal priors centered at zero with a standard deviation of 15 × om.scale, and the between-study heterogeneity standard deviation was assigned a uniform prior ranging from 0 to om.scale. The value of om.scale was automatically determined by gemtc according to the observed scale of each outcome network. Three Markov chains were run, with 20,000 iterations discarded as burn-in, followed by 100,000 sampling iterations per chain and a thinning interval of 1. Posterior treatment effects were reported as HRs or RRs with 95% credible intervals (CrIs). Convergence was evaluated using trace and density plots and the Gelman–Rubin statistic. Model fit was examined using residual deviance and the deviance information criterion (DIC). No network contained a closed loop; formal loop-specific inconsistency testing was therefore not possible, and comparisons of consistency and unrelated-effects models were treated as exploratory. Posterior rank probabilities and surface under the cumulative ranking curve (SUCRA) values were calculated as descriptive probabilistic summaries (35). Higher SUCRA values indicate a greater posterior probability of being among the more effective interventions but do not establish a treatment hierarchy. The main text identifies the intervention with the largest SUCRA value for each outcome and subgroup; these summaries are interpreted together with effect estimates, 95% CrIs, certainty of evidence, network sparsity, and the absence of direct NIC-versus-PIC comparisons. Complete rankings are provided in the **Supplementary Material**. Funnel plots were generated in Stata 17.0 when sufficient comparisons were available (36).

### 2.6 Certainty of evidence

Certainty of evidence for each network estimate was evaluated using GRADE within the CINeMA framework (37–39). Assessments considered within-study bias, reporting bias, indirectness, imprecision, heterogeneity, and incoherence. Minimum important differences were prespecified as relative effects of 1.25 for EFS, OS, and pCR and 1.20 for MPR (40). Evidence was rated as high, moderate, low, or very low. Risk-of-bias judgments from the separate RoB 2 assessment were incorporated into the within-study bias domain.

## 3 Results

### 3.1 Literature search and study selection

**Figure 1:**
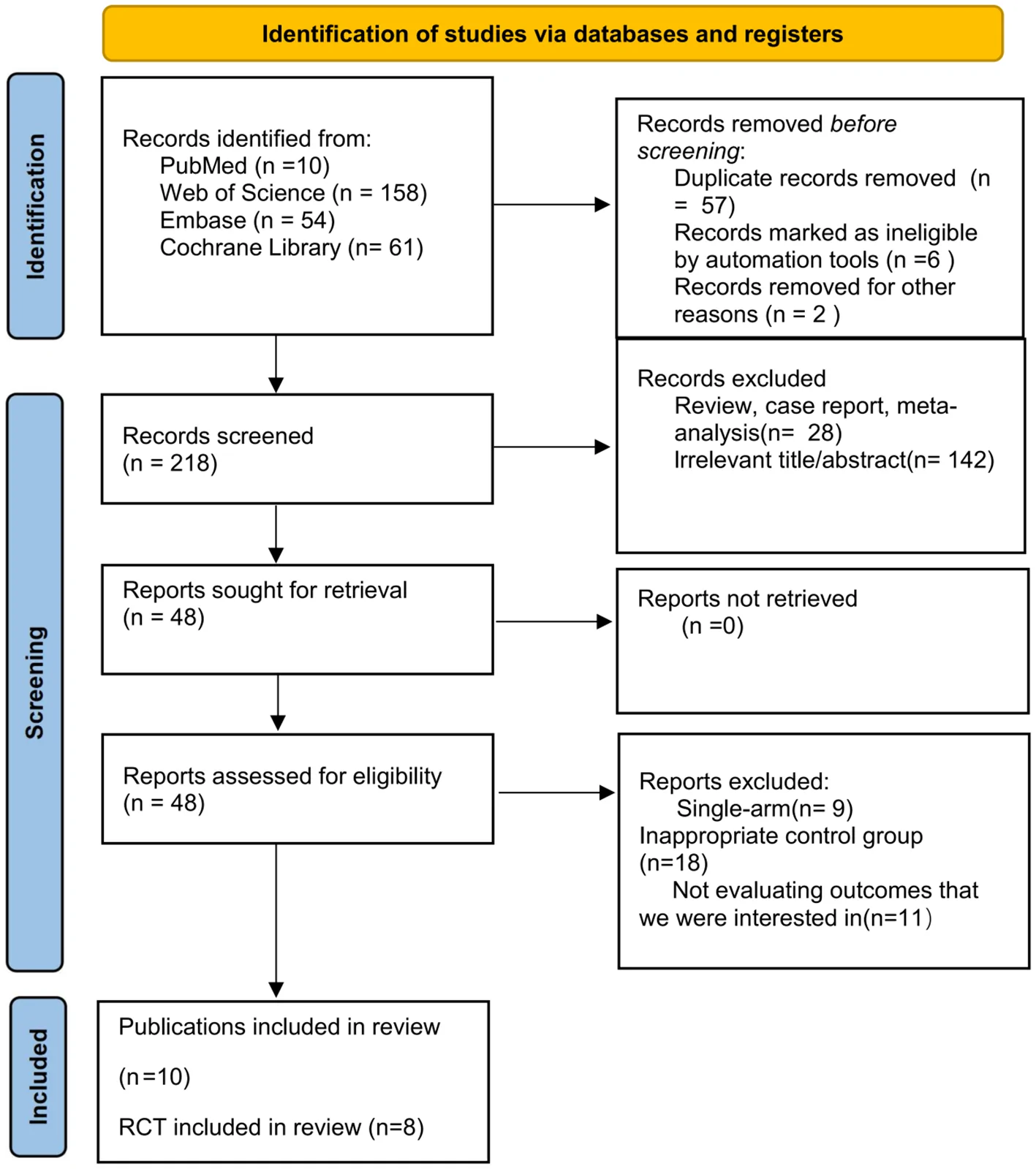
PRISMA flow diagram of the literature search and study selection process.

The database searches identified 283 records, all of which were imported into EndNote for reference management. Before screening, 57 duplicate records were removed, 6 records were marked as ineligible by automation tools, and 2 records were removed for other reasons, leaving 218 records for title and abstract screening. Of these, 170 records were excluded. All 48 reports sought for retrieval were successfully obtained and assessed for eligibility. Thirty-eight reports were excluded because they were single-arm or nonrandomized studies (n = 9), used an ineligible intervention or comparator (n = 18), or did not report outcomes relevant to this review (n = 11). Ultimately, 10 reports describing eight randomized controlled trials were included in the systematic review and network meta-analysis (9–11, 16–22) (**Figure 1**).

### 3.2 Study characteristics

The 10 included publications represented eight multicenter randomized controlled trials published between 2022 and 2025. The trials used open-label or double-blind designs and enrolled patients with stage IB–IIIB resectable NSCLC. Sample sizes and randomization ratios varied across trials, and median participant ages ranged from 61 to 66 years. Most participants were male. Histologic subtypes included squamous and non-squamous NSCLC, including adenocarcinoma, adenosquamous carcinoma, large-cell carcinoma, and undifferentiated carcinoma. PD-L1 expression was assessed using the tumor proportion score.

The evaluated immune checkpoint inhibitors included nivolumab, pembrolizumab, durvalumab, toripalimab, tislelizumab, and camrelizumab; nivolumab plus ipilimumab was the only dual-checkpoint regimen. Control groups received protocol-specified platinum-based chemotherapy, with matched placebo administered in blinded trials where applicable. Detailed study characteristics, PD-L1 subgroup data, and treatment schedules are presented in **Tables 1** and **2** and **Supplementary Tables 3**–**6**, **11**.

**Table 1:** Baseline characteristics of studies included in the network meta-analysis.

First author	Year	Phase/design	Sample size (intervention/control)	Stage	Histology	Strategy	Intervention Arm	Control Arm
Forde PM (9)	2022	Phase III, open-label	179/179	IB–IIIA	Non-squamous, 49.2%; squamous, 50.8%	NIC	Nivolumab + CT	CT
Awad MM (16)	2025	Phase III, open-label	113/108	IB–IIIA	Non-squamous, 51.6%; squamous, 48.4%	NIC	Nivolumab + ipilimumab	CT
Wakelee H (21)	2023	Phase III, double-blind	397/400	II–IIIB (N2)	Non-squamous, 56.8%; squamous, 43.2%	PIC	Pembrolizumab + CT	CT
Spicer JD (10)	2024	Phase III, double-blind	397/400	II–IIIB (N2)	Non-squamous, 56.8%; squamous, 43.2%	PIC	Pembrolizumab + CT	CT
Heymach JV (17)	2023	Phase III, double-blind	366/374	IIA–IIIB (N2)	Non-squamous, 50.7%; squamous, 48.7%	PIC	Durvalumab + CT	CT
Lu S (19)	2024	Phase III, double-blind	202/202	II–III	Non-squamous, 22.3%; squamous, 77.7%	PIC	Toripalimab + CT	CT
Yue DS (22)	2024	Phase III, double-blind	226/227	II–IIIA	Squamous, 78.1%; non-squamous, 21.0%; other, 0.9%	PIC	Tislelizumab + CT	CT
Provencio M (20)	2023	Phase II, open-label	57/29	IIIA–IIIB	Squamous, 40.7%; adenocarcinoma, 41.9%; other, 17.4%	PIC	Nivolumab + CT	CT
Lei J (18)	2023	Phase II, open-label	43/45	IIIA–IIIB	Squamous, 67.0%; adenocarcinoma, 29.5%; other, 3.4%	NIC	Camrelizumab + CT	CT
Cascone T (11)	2024	Phase III, double-blind	229/232	IIA–IIIB	Non-squamous, 49.2%; squamous, 50.8%	PIC	Nivolumab + CT	CT

**Table 2:** Characteristics of included randomized controlled trials.

First author	PD-L1 detection	PD-L1<1% patients (%)		PD-L1 ≥1% patients (%)		PD-L1 1-49% patients (%)		PD-L1 ≥50% patients (%)		Reported outcomes
		Intervention n(%)	Control,n(%)	Intervention,n(%)	Control,n(%)	Intervention,n(%)	Control,n(%)	Intervention,n(%)	Control,n(%)	
Forde PM (9)	TPS	78(43.6)	77(43.0)	89(49.7)	89(49.7)	51(28.5)	47(26.3)	38 (21.2)	42 (23.5)	EFS、pCR、MPR
Awad MM (16)	TPS	49 (43)	43 (40)	60 (53)	58 (54)	37 (33)	40 (37)	23 (20)	18 (17)	EFS、pCR、OS
Wakelee H (21)	TPS	138 (34.8)	151 (37.8)	/	/	127 (32.0)	115 (28.8)	132 (33.2)	134 (33.5)	EFS
Spicer JD (10)	TPS	138 (35.0)	151 (38.0)	/	/	127 (32)	115 (29)	132 (33)	134 (34)	EFS、OS
Heymach JV (17)	TPS	122(33.3)	125(33.4)	/	/	135(36.9)	142(38)	109(29.8)	107(28.6)	EFS、 pCR、MPR
Lu S (19)	TPS	51 (25.3)	54 (26.7)	/	/	69 (34.2)	68 (33.7)	64 (31.7)	64 (31.7)	EFS
Yue DS (22)	TPS	89 (39)	84 (37)	130 (58)	132 (58)	59 (26)	70 (31)	71 (31)	62 (27)	EFS、pCR、MPR
Provencio M (20)	TPS	20(35.1)	/	30(52.6)	18(65.2)	/	/	18(31.6)	/	pCR、OS
Lei J (18)	TPS	7 (16.3)	8 (17.8)	16 (37.2)	11(24.4)	/	/	/	/	pCR、MPR
Cascone T (11)	TPS	93 (40.6)	93 (40.1)	128 (55.9)	128 (55.2)	83 (36.2)	76 (32.8)	45 (19.7)	52 (22.4)	EFS、pCR、MPR

### 3.3 Risk-of-bias assessment

Among the 10 included publications, 6 were judged to have a low overall risk of bias and 4 were judged to have some concerns; none was judged to have a high overall risk. The four judgments of some concerns arose from the missing-outcome-data domain (**Supplementary Figure 1**).

### 3.4 Evidence structure and model diagnostics

None of the outcome networks contained a closed loop; therefore, loop-specific inconsistency could not be assessed. DIC values were similar between the consistency and unrelated-effects models across most outcome networks (**Supplementary Table 9**). Gelman–Rubin statistics approached 1.00, and the convergence and trace plots supported satisfactory chain convergence (**Supplementary Figures 2**–**25**).

### 3.5 Efficacy outcomes

#### 3.5.1 EFS

**Figure 2:**
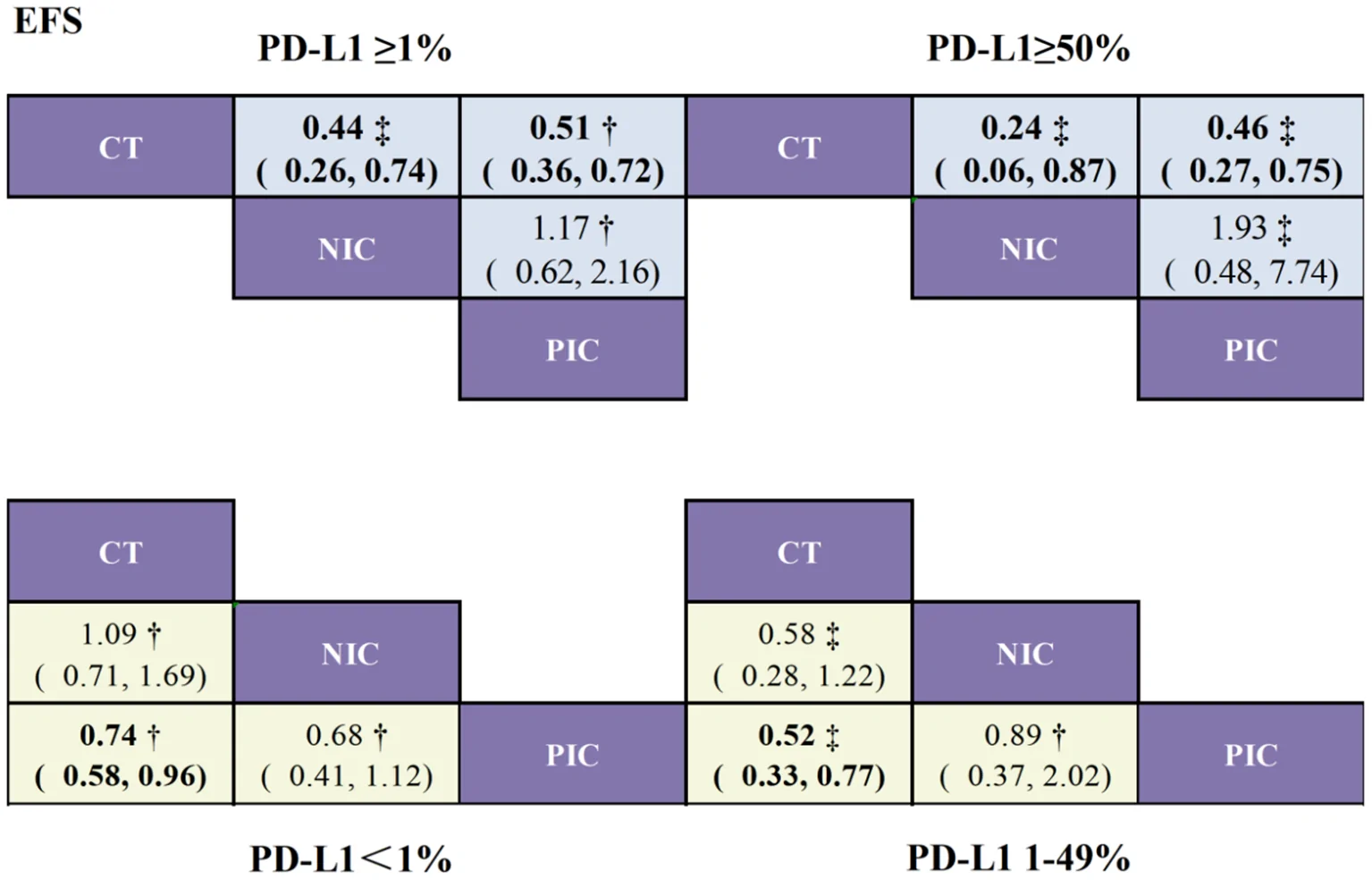
Bayesian network meta-analysis league table for EFS by PD-L1 subgroup. HRs below 1 favor the row-defining intervention. Certainty symbols are defined as follows: *, high; †, moderate; ‡, low; §, very low. CT, chemotherapy; EFS, event-free survival; HR, hazard ratio; NIC, neoadjuvant immunotherapy-based strategy; PIC, perioperative immunotherapy-based strategy.

In the PD-L1 <1% subgroup, PIC was associated with longer EFS than CT (HR 0.74, 95% CrI 0.58–0.96; moderate certainty) and had the largest SUCRA value in the corresponding network (96.3%) (**Figure 2**; **Supplementary Table 7**). In the PD-L1 ≥1% subgroup, PIC (HR 0.51, 95% CrI 0.36–0.72; moderate certainty) and NIC (HR 0.44, 95% CrI 0.26–0.74; low certainty) were associated with longer EFS than CT; NIC had the largest SUCRA value in that network (85.7%). In the PD-L1 1–49% subgroup, PIC was associated with longer EFS than CT (HR 0.52, 95% CrI 0.33–0.77; low certainty) and had the largest SUCRA value in that network (80.9%). In the PD-L1 ≥50% subgroup, NIC was associated with longer EFS than CT (HR 0.24, 95% CrI 0.06–0.87; low certainty) and had the largest SUCRA value in that network (91.4%).

The interventions with the largest SUCRA values were PIC for PD-L1 <1% and 1–49% and NIC for PD-L1 ≥1% and ≥50% (**Table 3**). These descriptive probabilistic summaries do not establish superiority, particularly because the networks lacked direct NIC-versus-PIC comparisons and several estimates were informed by sparse or low-certainty evidence. Complete EFS estimates and rankings are provided in **Figure 2** and **Supplementary Table 7**.

**Table 3:** SUCRA summary by outcome and PD-L1 subgroup.

Outcome	PD-L1 subgroup	Intervention with largest SUCRA	SUCRA (%)	Effect versus CT (95% CrI)	Certainty
EFS	<1%	PIC	96.3	HR 0.74 (0.58–0.96)	Moderate
EFS	≥1%	NIC	85.7	HR 0.44 (0.26–0.74)	Low
EFS	1–49%	PIC	80.9	HR 0.52 (0.33–0.77)	Low
EFS	≥50%	NIC	91.4	HR 0.24 (0.06–0.87)	Low
OS	<1%	PIC	73.9	HR 0.93 (0.65–1.33)	High
OS	≥1%	NIC	84.4	HR 0.47 (0.25–0.88)	Moderate
pCR	<1%	Camrelizumab + CT	83.1	RR 7.48 (1.83–55.23)	Moderate
pCR	≥1%	Tislelizumab + CT	85.8	RR 11.07 (5.35–27.31)	Moderate
pCR	1–49%	Nivolumab + CT	87.2	RR 8.58 (4.47–18.70)	High
pCR	≥50%	Durvalumab + CT	97.5	RR 6.34 (3.45–12.93)	Very low
MPR	<1%	Camrelizumab + CT	79.2	RR 3.76 (1.42–11.30)	Moderate
MPR	≥1%	Camrelizumab + CT	84.7	RR 8.28 (3.58–21.94)	Low

SUCRA values are descriptive and do not establish treatment superiority. They should be interpreted alongside effect estimates, 95% CrIs, certainty of evidence, and network limitations. Complete rankings are provided in **Supplementary Tables 7**, **8**.

#### 3.5.2 OS

**Figure 3:**
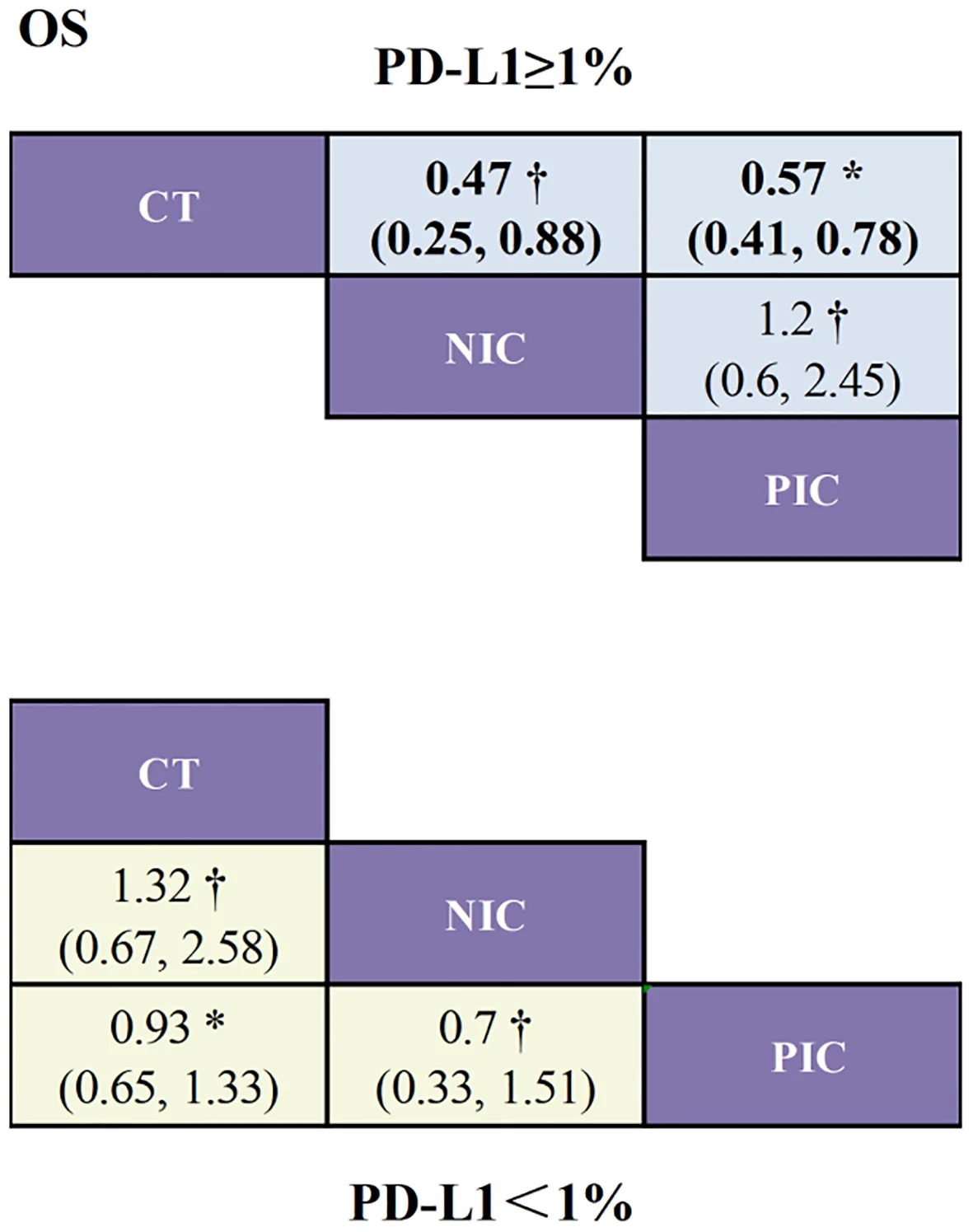
Bayesian network meta-analysis league table: Comparison of immunotherapy and chemotherapy for OS in NSCLC by PD-L1 expression (HRs below 1 favor the row-defining intervention).

OS analyses included three studies in each of the PD-L1 <1% and ≥1% subgroup networks. In the PD-L1 <1% subgroup, neither PIC (HR 0.93, 95% CrI 0.65–1.33; high certainty) nor NIC (HR 1.32, 95% CrI 0.67–2.58; moderate certainty) was associated with improved OS compared with CT. PIC had the largest SUCRA value in this subgroup network (73.9%) (**Figure 3**; **Table 3**; **Supplementary Table 7**).

In the PD-L1 ≥1% subgroup, PIC (HR 0.57, 95% CrI 0.41–0.78; high certainty) and NIC (HR 0.47, 95% CrI 0.25–0.88; moderate certainty) were associated with improved OS compared with CT. NIC had the largest SUCRA value in this network (84.4%) (**Figure 3**; **Table 3**; **Supplementary Table 7**). Each OS subgroup network included only three studies and no direct NIC-versus-PIC comparison; consequently, the SUCRA values are descriptive and do not establish superiority.

#### 3.5.3 pCR

Several preoperative immunotherapy-based regimens were associated with higher pCR than CT, with variation in the intervention that had the largest SUCRA value across PD-L1 subgroups. In the PD-L1 <1% subgroup, camrelizumab plus CT (RR 7.48, 95% CrI 1.83–55.23; moderate certainty), durvalumab plus CT (RR 2.58, 95% CrI 1.22–6.01; high certainty), nivolumab plus CT (RR 3.89, 95% CrI 2.21–7.37; high certainty), nivolumab plus ipilimumab (RR 3.40, 95% CrI 1.30–10.78; moderate certainty), and tislelizumab plus CT (RR 4.89, 95% CrI 2.34–11.48; moderate certainty) were associated with higher pCR than CT. Camrelizumab plus CT had the largest SUCRA value in this network (83.1%). In the PD-L1 ≥1% subgroup, tislelizumab plus CT (RR 11.07, 95% CrI 5.35–27.31; moderate certainty), nivolumab plus ipilimumab (RR 5.05, 95% CrI 2.32–12.80; low certainty), durvalumab plus CT (RR 9.77, 95% CrI 6.25–16.20; high certainty), and camrelizumab plus CT (RR 4.22, 95% CrI 1.60–13.32; moderate certainty) were associated with higher pCR than CT. Tislelizumab plus CT had the largest SUCRA value in this network (85.8%).

**Figure 4:**
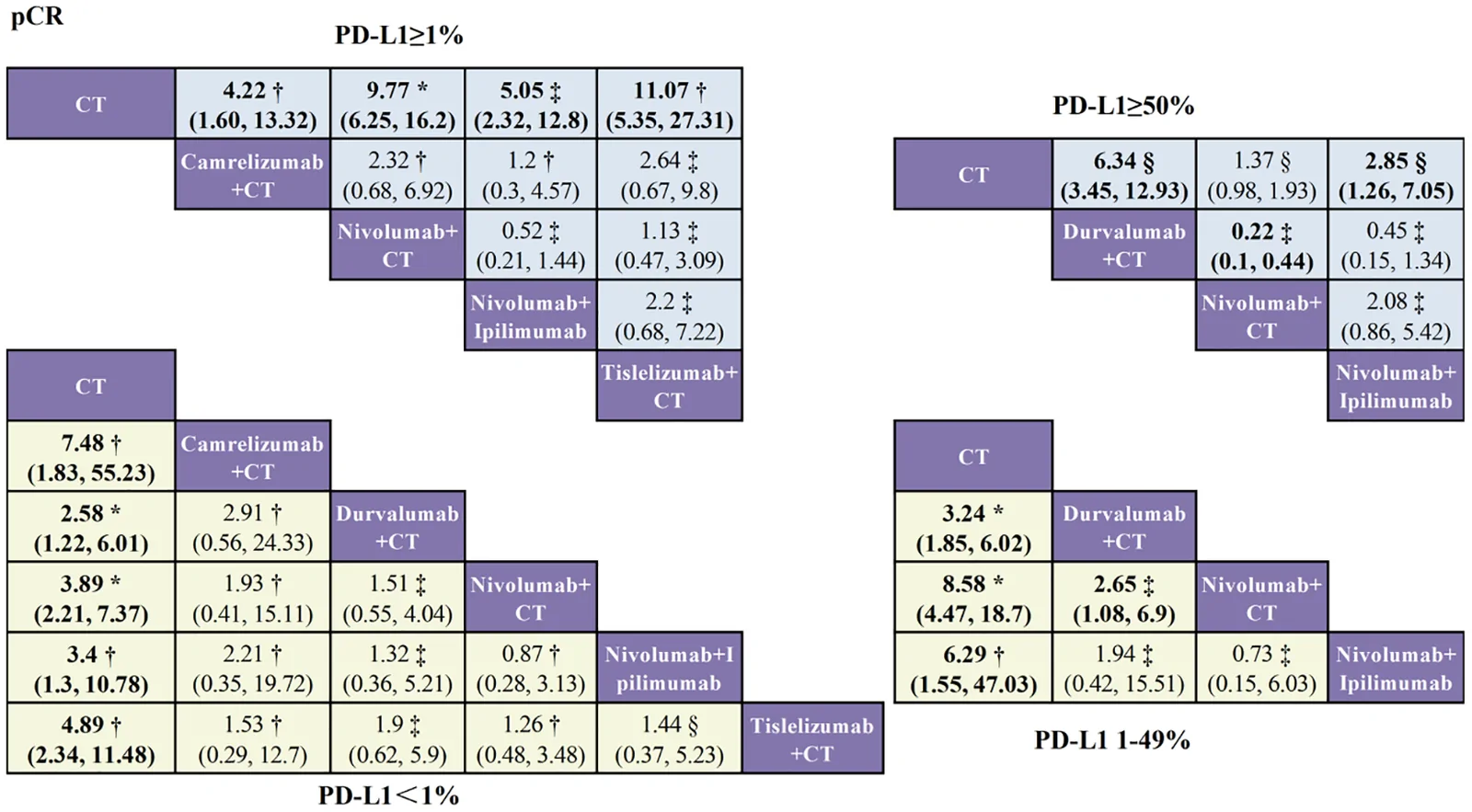
Bayesian network meta-analysis league table: Comparison of immunotherapy versus chemotherapy for pCR in NSCLC patients, stratified by PD-L1 expression level (RRs above 1 favor the row-defining intervention).

In the PD-L1 1–49% subgroup, durvalumab plus CT (RR 3.24, 95% CrI 1.85–6.02; high certainty), nivolumab plus CT (RR 8.58, 95% CrI 4.47–18.70; high certainty), and nivolumab plus ipilimumab (RR 6.29, 95% CrI 1.55–47.03; moderate certainty) were associated with higher pCR than CT. Nivolumab plus CT had the largest SUCRA value in this network (87.2%). In the PD-L1 ≥50% subgroup, durvalumab plus CT (RR 6.34, 95% CrI 3.45–12.93; very low certainty) and nivolumab plus ipilimumab (RR 2.85, 95% CrI 1.26–7.05; very low certainty) were associated with higher pCR than CT; the estimate for nivolumab plus CT included the null (RR 1.37, 95% CrI 0.98–1.93). Durvalumab plus CT had the largest SUCRA value in this network (97.5%). The corresponding interventions and SUCRA values are summarized descriptively in **Table 3**; complete estimates and rankings are provided in **Supplementary Table 8**. These SUCRA summaries should be interpreted cautiously because several subgroup networks were sparse and certainty was very low for some comparisons (**Figure 4**; **Supplementary Figure 32**; **Supplementary Table 8**).

#### 3.5.4 MPR

**Figure 5:**
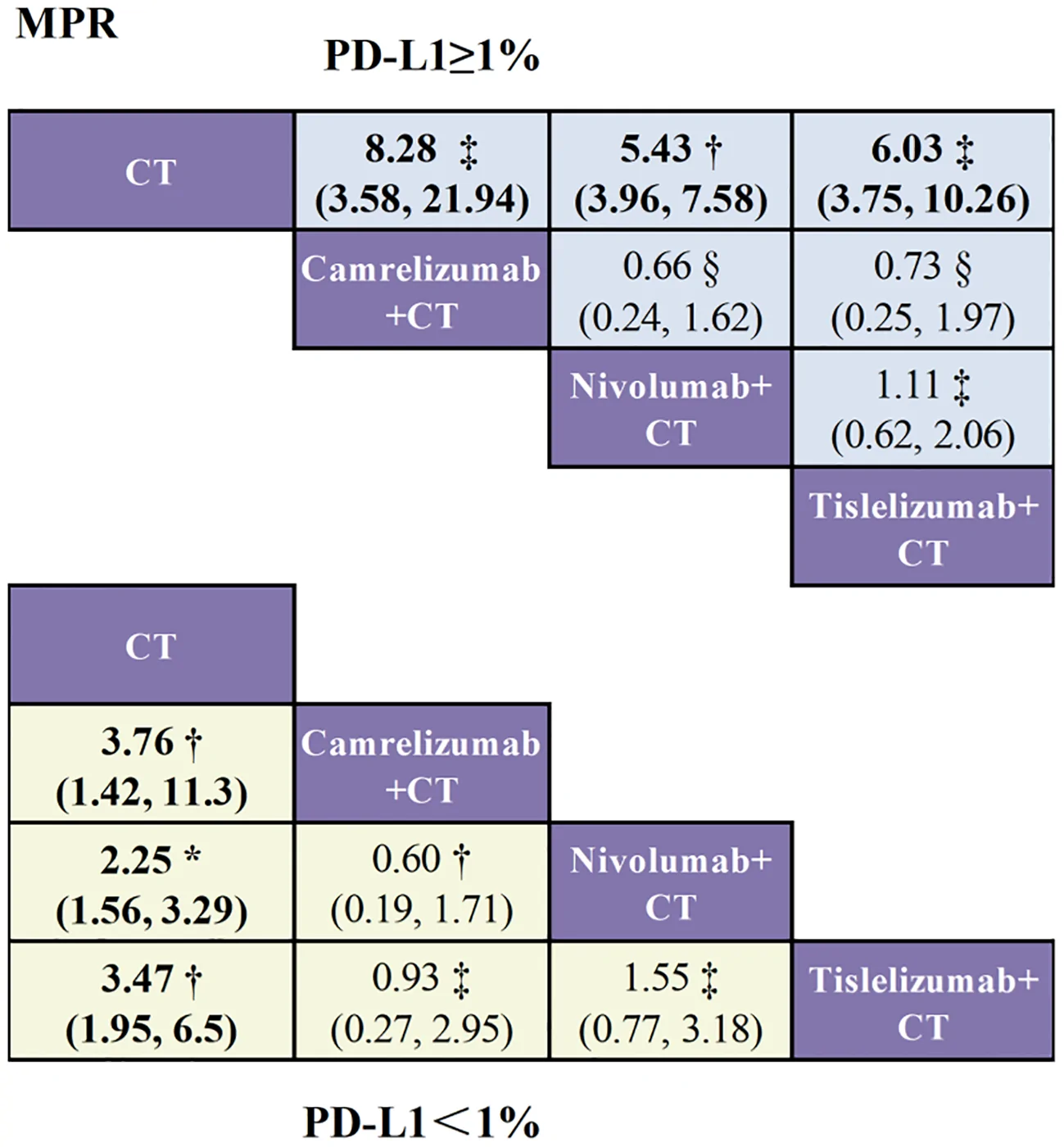
Bayesian network meta-analysis league table: Comparison of immunotherapy and chemotherapy for MPR in NSCLC by PD-L1 expression (RRs above 1 favor the row-defining intervention).

In the PD-L1 <1% subgroup, camrelizumab plus CT (RR 3.76, 95% CrI 1.42–11.30; moderate certainty), nivolumab plus CT (RR 2.25, 95% CrI 1.56–3.29; high certainty), and tislelizumab plus CT (RR 3.47, 95% CrI 1.95–6.50; moderate certainty) were associated with higher MPR than CT. Camrelizumab plus CT had the largest SUCRA value in this network (79.2%). In the PD-L1 ≥1% subgroup, camrelizumab plus CT (RR 8.28, 95% CrI 3.58–21.94; low certainty), nivolumab plus CT (RR 5.43, 95% CrI 3.96–7.58; moderate certainty), and tislelizumab plus CT (RR 6.03, 95% CrI 3.75–10.26; low certainty) were associated with higher MPR than CT. Camrelizumab plus CT had the largest SUCRA value in this network (84.7%) (**Figure 5**; **Table 3**; **Supplementary Figure 32**; **Supplementary Table 8**).

No statistically credible differences were observed among active preoperative regimens. Complete MPR estimates and rankings are provided in **Supplementary Table 8**. The SUCRA ordering therefore does not establish superiority among active regimens.

### 3.6 Heterogeneity and publication bias

I² values ranged from 0% to 67% across outcomes and PD-L1 subgroups (**Supplementary Table 9**). EFS I² values ranged from 0% in PD-L1 <1% to 22% in PD-L1 ≥50%, and OS values ranged from 8% in PD-L1 <1% to 67% in PD-L1 ≥1%. For pathologic outcomes, pCR I² values ranged from 5% in PD-L1 ≥1% to 45.2% in PD-L1 ≥50%, and MPR values ranged from 0.3% in PD-L1 <1% to 46% in PD-L1 ≥1%. Funnel plots for pCR and MPR did not show marked asymmetry or extreme clustering (**Supplementary Figures 26**–**31**). The number of contributing studies was small in several networks, limiting the informativeness of visual assessment.

### 3.7 Certainty of evidence

GRADE-CINeMA assessments classified 8 comparisons as high certainty, 31 as moderate certainty, 22 as low certainty, and 6 as very low certainty (**Supplementary Table 10**). The high-certainty comparisons comprised 2 OS, 5 pCR, and 1 MPR comparison; the remaining distributions by outcome are reported in **Supplementary Table 10**.

## 4 Discussion

This PD-L1-stratified network meta-analysis found that the relative effects of neoadjuvant and perioperative immunotherapy-based strategies varied across subgroups and endpoints. PIC was associated with improved EFS in patients with PD-L1 expression <1%, whereas both PIC and NIC were associated with improved EFS and OS in PD-L1-positive disease. The preoperative regimens associated with the most favorable pathologic outcomes differed across PD-L1 strata. These findings, however, were based primarily on indirect comparisons and do not establish the superiority of any active strategy.

The findings are broadly consistent with CheckMate 816, KEYNOTE-671, AEGEAN, CheckMate 77T, Neotorch, and RATIONALE-315, which demonstrated EFS or pathologic-response benefits for immunotherapy-based treatment compared with chemotherapy (9–11, 16, 17, 19, 21, 41). A previous indirect meta-analysis by Zhou et al. evaluated the incremental value of postoperative immunotherapy and did not identify a statistically significant EFS or OS advantage for adding an adjuvant phase (42). The present analysis addressed a different question by examining trial-defined PD-L1 subgroups and separating strategy-level survival outcomes from regimen-level pathologic outcomes.

Several biological hypotheses may account for the observed subgroup patterns, although none can be tested by this study-level meta-analysis. Treatment delivered while the tumor remains *in situ* may enhance antigen release and T-cell priming, whereas postoperative immune therapy could help sustain control of residual disease (24, 26). Greater PD-L1 expression may identify tumors with more active PD-1-mediated immune suppression, but PD-L1 remains an imperfect and dynamic marker. PD-1 and CTLA-4 blockade also act through distinct, potentially complementary mechanisms (27, 28). The PD-L1-stratified estimates for nivolumab plus ipilimumab should therefore be interpreted as possible modification of a PD-1-containing combination rather than evidence that PD-L1 predicts ipilimumab activity.

This study has several strengths, including the incorporation of recent randomized evidence, evaluation of clinically relevant PD-L1 subgroups, separation of survival and pathologic outcomes according to treatment timing, and integration of Bayesian network estimates with GRADE-CINeMA certainty assessments. Presenting SUCRA values alongside relative effects, 95% CrIs, and certainty ratings also allowed the ranking results to be interpreted within the broader evidence context.

Several limitations should be acknowledged. The absence of closed loops and direct NIC-versus-PIC comparisons made the analysis dependent on the transitivity assumption, while differences in disease stage, treatment duration, postoperative therapy, surgical completion, and PD-L1 subgroup reporting may have introduced residual indirectness. Some subgroup analyses were based on limited data, several CrIs were wide, and the dual-checkpoint estimate was derived from a single exploratory randomized comparison. Safety, surgical delay, immune-related adverse events, and adherence to postoperative therapy were not synthesized, and the use of study-level subgroup data precluded conclusions for individual patients. The present findings should therefore be considered hypothesis-generating, and prospective head-to-head trials using standardized PD-L1 assays and subgroup definitions, mature OS follow-up, and integrated efficacy, safety, surgical, and quality-of-life outcomes are warranted.

## Conclusion

PD-L1-stratified indirect evidence suggests that the relative effects of neoadjuvant and perioperative immunotherapy-based strategies may vary across subgroups and endpoints, but does not establish the superiority of either approach. Prospective head-to-head trials using standardized PD-L1 assays and subgroup definitions, mature survival follow-up, and integrated efficacy and safety outcomes are needed.

## Data Availability

The original contributions presented in the study are included in the article/**Supplementary Material**. Further inquiries can be directed to the corresponding author.
