# Supplementary material for: Neoadjuvant versus perioperative immunotherapy in resectable NSCLC: A PD-L1–stratified Bayesian network meta-analysis
Source: Front Oncol. 2026 Aug 6;16:1905730. doi: 10.3389/fonc.2026.1905730 (PMC13489855; doi:10.3389/fonc.2026.1905730)
Supplement: Supplementary file 1 [file DataSheet1.docx]

Neoadjuvant versus Perioperative Immunotherapy in Resectable NSCLC: A PD-L1–Stratified Bayesian Network Meta-Analysis

| **Table of Contents** | | |
| --- | --- | --- |
| Title | Content | page |
| Table S1 | PRISMA NMA Checklist of Items to Include When Reporting a Systematic Review Involving a Network Meta-analysis | 3-7 |
| Table S2 | Literature Search Strategy | 8-11 |
| Table S3 | Characteristics of Included Randomized Controlled Trials: PD-L1 Expression-Stratified Event-Free Survival （EFS）Hazard Ratios | 12 |
| Table S4 | Characteristics of Included Randomized Controlled Trials: PD-L1 Expression-Stratified Overall Survival (OS) Hazard Ratios | 13 |
| Table S5 | Characteristics of Included Randomized Controlled Trials: PD-L1 Expression-Stratified Pathological Complete Response (pCR) Data | 14 |
| Table S6 | Characteristics of Included Randomized Controlled Trials: PD-L1 Expression-Stratified Major Pathological Response (MPR) Data | 15 |
| Table S7 | SUCRA-Based Ranking of Treatment Regimens for Efficacy Endpoints in the Bayesian Network Meta-Analysis | 16 |
| Table S8 | SUCRA-Based Ranking of Treatment Regimens for Efficacy Endpoints in the Bayesian Network Meta-Analysis | 17 |
| Table S9 | Meta-Analysis Outcomes of EFS, OS, pCR, and MPR by PD-L1 Expression Status | 18 |
| Table S10 | Distribution of evidence quality grades for each clinical endpoint across different PD-L1 expression subgroups | 19 |
| Table S11 | Detailed Treatment Regimens of the Included Randomized Controlled Trials | 20-22 |
| Figure S1 | Summary of results from assessment of studies by using Cochrane Risk of Bias Tool 2.0 | 23 |
| Figure S2 | Convergence diagnostics of Bayesian network meta-analysis for EFS in NSCLC with PD-L1 expression < 1% | 24 |
| Figure S3 | Bayesian network meta-analysis of EFS in NSCLC with PD-L1 expression ≥ 1% | 25 |
| Figure S4 | Bayesian network meta-analysis of EFS in NSCLC with PD-L1 expression 1–49% | 26 |
| Figure S5 | Bayesian network meta-analysis of EFS in NSCLC with PD-L1 expression ≥ 50% | 27 |
| Figure S6 | Convergence diagnostics of Bayesian network meta-analysis for OS in NSCLC with PD-L1 expression < 1% | 28 |
| Figure S7 | Convergence diagnostic plots of Bayesian network meta-analysis for OS in NSCLC with PD-L1 expression ≥ 1% | 29 |
| Figure S8 | Convergence diagnostics of Bayesian network meta-analysis for pCR in NSCLC with PD-L1 expression < 1% | 30 |
| Figure S9 | Convergence diagnostics of Bayesian network meta-analysis for pCR in NSCLC with PD-L1 expression ≥ 1% | 31 |
| Figure S10 | Convergence diagnostics of Bayesian network meta-analysis for pCR in NSCLC with PD-L1 expression 1–49% | 32 |
| Figure S11 | Convergence diagnostics of Bayesian network meta-analysis for pCR in NSCLC withPD-L1 expression ≥ 50% | 33 |
| Figure S12 | Convergence diagnostics of Bayesian network meta-analysis for MPR in NSCLC with PD-L1 expression < 1% | 34 |
| Figure S13 | Convergence diagnostics of Bayesian network meta-analysis for MPR in NSCLC with PD-L1 expression ≥ 1% | 35 |
| Figure S14 | Trace plots and density plots of Bayesian network meta-analysis for EFS in NSCLC with PD-L1 expression < 1% | 36 |
| Figure S15 | Trace plots and density plots of Bayesian network meta-analysis for EFS in NSCLC with PD-L1 expression ≥ 1% | 37 |
| Figure  S16 | Trace plots and density plots of Bayesian network meta-analysis for EFS in NSCLC with PD-L1 expression 1–49% | 38 |
| Figure S17 | Trace plots and density plots of Bayesian network meta-analysis for EFS in NSCLC with PD-L1 expression ≥ 50% | 39 |
| Figure S18 | Trace plots and density plots of Bayesian network meta-analysis for OS in NSCLC with PD-L1 expression < 1% | 40 |
| Figure S19 | Trace plots and density plots of Bayesian network meta-analysis for OS in NSCLC with PD-L1 expression ≥ 1% | 41 |
| Figure S20 | Trace plots and density plots of Bayesian network meta-analysis for pCR in NSCLC with PD-L1 expression < 1% | 42 |
| Figure S21 | Trace plots and density plots of Bayesian network meta-analysis for pCR in NSCLC with PD-L1 expression ≥ 1% | 43 |
| Figure S22 | Trace plots and density plots of Bayesian network meta-analysis for pCR in NSCLC with PD-L1 expression 1–49% | 44 |
| Figure S23 | Trace plots and density plots of Bayesian network meta-analysis for pCR in NSCLC with PD-L1 expression ≥ 50% | 45 |
| Figure S24 | Trace plots and density plots of Bayesian network meta-analysis for MPR in NSCLC with PD-L1 expression < 1% | 46 |
| Figure S25 | Trace plots and density plots of Bayesian network meta-analysis for MPR in NSCLC with PD-L1 expression ≥ 1% | 47 |
| Figure S26 | Funnel plot for publication bias assessment of pCR in non-small cell lung cancer patients with PD-L1 expression < 1% | 48 |
| Figure S27 | Funnel plot for publication bias assessment of pCR in non-small cell lung cancer patients with PD-L1 expression ≥ 1% | 49 |
| Figure S28 | Funnel plot for publication bias assessment of pCR in non-small cell lung cancer patients with PD-L1 expression 1~49% | 50 |
| Figure S29 | Funnel plot for publication bias assessment of pCR in non-small cell lung cancer patients with PD-L1 expression ≥50% | 51 |
| Figure S30 | Funnel plot for publication bias assessment of MPR in non-small cell lung cancer patients with PD-L1 expression ＜1% | 52 |
| Figure S31. | Funnel plot for publication bias assessment of MPR in non-small cell lung cancer patients with PD-L1 expression≥1% | 53 |
| Figure S32 | Network graphs of comparative interventions for pCR and MPR in patients with NSCLC | 54 |

Table S1	PRISMA NMA Checklist of Items to Include When Reporting a Systematic Review Involving a Network Meta-analysis

| **Section/Topic** | **Item#** | **Checklist Item** | **Reported on Page #** |
| --- | --- | --- | --- |
| **TITLE** |  |  |  |
| Title | 1 | Identify the report as a systematic review *incorporating*  *anetwork meta-analysis (or related form of meta-analysis).* | 1 |
|  |  |  |  |
| **ABSTRACT** |  |  |  |
| Structured summary | 2 | Provide a structured summary including, as applicable:  **Background:** main objectives  **Methods:** data sources; study eligibility criteria, participants, and interventions; study appraisal; and *synthesis methods, such as network meta-analysis.*  **Results:** number of studies and participants identified; summary estimates with corresponding confidence/credible intervals; *treatment rankings may also be discussed. Authors may choose to summarize pairwise comparisons against a chosen treatment included in their analyses for brevity.*  **Discussion/Conclusions:** limitations; conclusions and implications of findings.  **Other:** systematic review registration number with registry name. | 1-2 |
|  |  |  |  |
| **INTRODUCTION** |  |  |  |
| Rationale | 3 | Describe the rationale for the review in the context of what is already known*, including mention of why a network meta-analysis has been conducted.* | 2 |
| Objectives | 4 | Provide an explicit statement of questions being addressed, with reference to participants, interventions, comparisons, outcomes, and study design (PICOS). | 2-3 |
|  |  |  |  |
| **METHODS** |  |  |  |
| Protocol and registration | 5 | Indicate whether a review protocol exists and if and where it can be accessed (e.g., Web address); and, if available, provide registration information, including registration number. | 3 |
| Eligibility criteria | 6 | Specify study characteristics (e.g., PICOS, length of follow-up) and report characteristics (e.g., years considered, language, publication status) used as criteria for eligibility, giving rationale. *Clearly describe eligible treatments included in the treatment network, and note whether any have been clustered or merged into the same node (with justification).* | 4 |
| Information sources | 7 | Describe all information sources (e.g., databases with dates of coverage, contact with study authors to identify additional studies) in the search and date last searched. | 3-4 |
| Search | 8 | Present full electronic search strategy for at least one database, including any limits used, such that it could be repeated. | 3-4  Supplementary  TableS2 |
| Study selection | 9 | State the process for selecting studies (i.e., screening, eligibility, included in systematic review, and, if applicable, included in the meta-analysis). | 4 |
| Data collection process | 10 | Describe method of data extraction from reports (e.g., piloted forms, independently, in duplicate) and any processes for obtaining and confirming data from investigators. | 4 |
| Data items | 11 | List and define all variables for which data were sought (e.g., PICOS, funding sources) and any assumptions and simplifications made. | 4 |
| **Geometry of the network** | **S1** | Describe methods used to explore the geometry of the treatment network under study and potential biases related to it. This should include how the evidence base has been graphically summarized for presentation, and what characteristics were compiled and used to describe the evidence base to readers. | 4 |
| Risk of bias within individual studies | 12 | Describe methods used for assessing risk of bias of individual studies (including specification of whether this was done at the study or outcome level), and how this information is to be used in any data synthesis. | 4 |
| Summary measures | 13 | State the principal summary measures (e.g., risk ratio, difference in means). *Also describe the use of additional summary measures assessed, such as treatment rankings and surface under the cumulative ranking curve (SUCRA) values, as well as modified approaches used to present summary findings from meta-analyses.* | 4 |
| Planned methods of analysis | 14 | Describe the methods of handling data and combining results of studies for each network meta-analysis. This should include, but not be limited to:   - *Handling of multi-arm trials;* - *Selection of variance structure;* - *Selection of prior distributions in Bayesian analyses; and* - *Assessment of model fit.* | 4-5 |
| **Assessment of Inconsistency** | **S2** | Describe the statistical methods used to evaluate the agreement of direct and indirect evidence in the treatment network(s) studied. Describe efforts taken to address its presence when found. | 4 |
| Risk of bias across studies | 15 | Specify any assessment of risk of bias that may affect the cumulative evidence (e.g., publication bias, selective reporting within studies). | 4 |
| Additional analyses | 16 | Describe methods of additional analyses if done, indicating which were pre-specified. This may include, but not be limited to, the following:   - Sensitivity or subgroup analyses; - Meta-regression analyses; - *Alternative formulations of the treatment network; and* - *Use of alternative prior distributions for Bayesian analyses (if applicable).* | 4-5 |
| **RESULTS†** |  |  |  |
| Study selection | 17 | Give numbers of studies screened, assessed for eligibility, and included in the review, with reasons for exclusions at each stage, ideally with a flow diagram. | 5,Fig1 |
| **Presentation of network structure** | **S3** | Provide a network graph of the included studies to enable visualization of the geometry of the treatment network. | 6,Fig2 |
| **Summary of network geometry** | **S4** | Provide a brief overview of characteristics of the treatment network. This may include commentary on the abundance of trials and randomized patients for the different interventions and pairwise comparisons in the network, gaps of evidence in the treatment network, and potential biases reflected by the network structure. | Fig 32 |
| Study characteristics | 18 | For each study, present characteristics for which data were extracted (e.g., study size, PICOS, follow-up period) and provide the citations. | 5,Table 1 |
| Risk of bias within studies | 19 | Present data on risk of bias of each study and, if available, any outcome level assessment. | 5-6,Figure S1 |
| Results of individual studies | 20 | For all outcomes considered (benefits or harms), present, for each study: 1) simple summary data for each intervention group, and 2) effect estimates and confidence intervals. *Modified approaches may be needed to deal with information from larger networks.* | 6-8 |
| Synthesis of results | 21 | Present results of each meta-analysis done, including confidence/credible intervals. *In larger networks, authors may focus on comparisons versus a particular comparator (e.g. placebo or standard care), with full findings presented in an appendix. League tables and forest plots may be considered to summarize pairwise comparisons.* If additional summary measures were explored (such as treatment rankings), these should also be presented. | 6-8 |
| **Exploration for inconsistency** | **S5** | Describe results from investigations of inconsistency. This may include such information as measures of model fit to compare consistency and inconsistency models, *P* values from statistical tests, or summary of inconsistency estimates from different parts of the treatment network. | 8 |
| Risk of bias across studies | 22 | Present results of any assessment of risk of bias across studies for the evidence base being studied. | 6-7 |
| Results of additional analyses | 23 | Give results of additional analyses, if done (e.g., sensitivity or subgroup analyses, meta-regression analyses*, alternative network geometries studied, alternative choice of prior distributions for Bayesian analyses,* and so forth). | 8-9 |
|  |  |  |  |
| **DISCUSSION** |  |  |  |
| Summary of evidence | 24 | Summarize the main findings, including the strength of evidence for each main outcome; consider their relevance to key groups (e.g., healthcare providers, users, and policy-makers). | 9-13 |
| Limitations | 25 | Discuss limitations at study and outcome level (e.g., risk of bias), and at review level (e.g., incomplete retrieval of identified research, reporting bias). *Comment on the validity of the assumptions, such as transitivity and consistency. Comment on any concerns regarding network geometry (e.g., avoidance of certain comparisons).* | 13 |
| Conclusions | 26 | Provide a general interpretation of the results in the context of other evidence, and implications for future research. | 1 |
|  |  |  |  |
| **FUNDING** |  |  |  |
| Funding | 27 | Describe sources of funding for the systematic review and other support (e.g., supply of data); role of funders for the systematic review. This should also include information regarding whether funding has been received from manufacturers of treatments in the network and/or whether some of the authors are content experts with professional conflicts of interest that could affect use of treatments in the network. | NA |

PICOS = population, intervention, comparators, outcomes, study design.

* Text in italics indicate S wording specific to reporting of network meta-analyses that has been added to guidance from the PRISMA statement.

† Authors may wish to plan for use of appendices to present all relevant information in full detail for items in this section.

| **Table S2.Literature Search Strategy** | |
| --- | --- |
| **Pubmed** | #1:Query："Carcinoma, Non-Small-Cell Lung"[Mesh] Results:81747  #2:Query: ((((((((((("Carcinoma, Non-Small-Cell Lung"[Mesh]) OR (Carcinoma, Non Small Cell Lung[Title/Abstract])) OR (Carcinomas, Non-Small-Cell Lung[Title/Abstract])) OR (Lung Carcinoma, Non-Small-Cell[Title/Abstract])) OR (Lung Carcinomas, Non-Small-Cell[Title/Abstract])) OR (Lung Carcinomas, Non-Small-Cell[Title/Abstract])) OR (Lung Carcinomas, Non-Small-Cell[Title/Abstract])) OR (Non-Small Cell Lung Cancer[Title/Abstract])) OR (Non-Small-Cell Lung Carcinoma[Title/Abstract])) OR (Non Small Cell Lung Carcinoma[Title/Abstract])) OR (Nonsmall Cell Lung Cancer[Title/Abstract])) OR (Non-Small Cell Lung Carcinoma[Title/Abstract]) Results:116998  #3:Query: "Neoadjuvant Therapy"[Mesh] Results:34780  #4:Query:(((((((((((((((((((((((((((((((((((((((((((((((("Neoadjuvant Therapy"[Mesh]) OR (Neoadjuvant Therapies[Title/Abstract])) OR (Therapy, Neoadjuvant[Title/Abstract])) OR (Neoadjuvant Treatment[Title/Abstract])) OR (Neoadjuvant Treatments[Title/Abstract])) OR (Treatment, Neoadjuvant[Title/Abstract])) OR (Neoadjuvant Chemotherapy[Title/Abstract])) OR (Chemotherapy, Neoadjuvant[Title/Abstract])) OR (Neoadjuvant Chemotherapies[Title/Abstract])) OR (Neoadjuvant Chemotherapy Treatment[Title/Abstract])) OR (Chemotherapy Treatment, Neoadjuvant[Title/Abstract])) OR (Neoadjuvant Chemotherapy Treatments[Title/Abstract])) OR (Treatment, Neoadjuvant Chemotherapy[Title/Abstract])) OR (Neoadjuvant Chemoradiotherapy[Title/Abstract])) OR (Chemoradiotherapy, Neoadjuvant[Title/Abstract])) OR (Neoadjuvant Chemoradiotherapies[Title/Abstract])) OR (Neoadjuvant Chemoradiation[Title/Abstract])) OR (Chemoradiation, Neoadjuvant[Title/Abstract])) OR (Neoadjuvant Chemoradiations[Title/Abstract])) OR (Neoadjuvant Chemoradiation Therapy[Title/Abstract])) OR (Chemoradiation Therapy, Neoadjuvant[Title/Abstract])) OR (Neoadjuvant Chemoradiation Therapies[Title/Abstract])) OR (Therapy, Neoadjuvant Chemoradiation[Title/Abstract])) OR (Neoadjuvant Chemoradiation Treatment[Title/Abstract])) OR (Chemoradiation Treatment, Neoadjuvant[Title/Abstract])) OR (Neoadjuvant Chemoradiation Treatments[Title/Abstract])) OR (Treatment, Neoadjuvant Chemoradiation[Title/Abstract])) OR (Neoadjuvant Systemic Therapy[Title/Abstract])) OR (Neoadjuvant Systemic Therapies[Title/Abstract])) OR (Systemic Therapy, Neoadjuvant[Title/Abstract])) OR (Therapy, Neoadjuvant Systemic[Title/Abstract])) OR (Neoadjuvant Systemic Treatment[Title/Abstract])) OR (Neoadjuvant Systemic Treatments[Title/Abstract])) OR (Systemic Treatment, Neoadjuvant[Title/Abstract])) OR (Treatment, Neoadjuvant Systemic[Title/Abstract])) OR (Neoadjuvant Radiotherapy[Title/Abstract])) OR (Neoadjuvant Radiotherapies[Title/Abstract])) OR (Radiotherapy, Neoadjuvant[Title/Abstract])) OR (Neoadjuvant Radiation[Title/Abstract])) OR (Neoadjuvant Radiations[Title/Abstract])) OR (Radiation, Neoadjuvant[Title/Abstract])) OR (Neoadjuvant Radiation Therapy[Title/Abstract])) OR (Neoadjuvant Radiation Therapies[Title/Abstract])) OR (Radiation Therapy, Neoadjuvant[Title/Abstract])) OR (Therapy, Neoadjuvant Radiation[Title/Abstract])) OR (Neoadjuvant Radiation Treatment[Title/Abstract])) OR (Neoadjuvant Radiation Treatments[Title/Abstract])) OR (Radiation Treatment, Neoadjuvant[Title/Abstract])) OR (Treatment, Neoadjuvant Radiation[Title/Abstract]) Results:55102  #5:Query: "Chemotherapy, Adjuvant"[Mesh] Results:50087  #6:Query: ((("Chemotherapy, Adjuvant"[Mesh]) OR (Adjuvant Chemotherapy[Title/Abstract])) OR (Adjuvant Drug Therapy[Title/Abstract])) OR (Drug Therapy, Adjuvant[Title/Abstract]) Results:72544  #7:Query:"Immune Checkpoint Inhibitors"[Mesh] Results:17883  #8:Query:(((((((((((((((((((((((((((((((("Immune Checkpoint Inhibitors"[Mesh]) OR (Checkpoint Inhibitors, Immune[Title/Abstract])) OR (Immune Checkpoint Blockers[Title/Abstract])) OR (Checkpoint Blockers, Immune[Title/Abstract])) OR (Immune Checkpoint Inhibitor[Title/Abstract])) OR (Checkpoint Inhibitor, Immune[Title/Abstract])) OR (CTLA-4 Inhibitors[Title/Abstract])) OR (CTLA 4 Inhibitors[Title/Abstract])) OR (Cytotoxic T-Lymphocyte-Associated Protein 4 Inhibitors[Title/Abstract])) OR (Cytotoxic T Lymphocyte Associated Protein 4 Inhibitors[Title/Abstract])) OR (Cytotoxic T-Lymphocyte-Associated Protein 4 Inhibitor[Title/Abstract])) OR (Cytotoxic T Lymphocyte Associated Protein 4 Inhibitor[Title/Abstract])) OR (CTLA-4 Inhibitor[Title/Abstract])) OR (CTLA 4 Inhibitor[Title/Abstract])) OR (PD-1 Inhibitors[Title/Abstract])) OR (PD 1 Inhibitors[Title/Abstract])) OR (Programmed Cell Death Protein 1 Inhibitor[Title/Abstract])) OR (Programmed Cell Death Protein 1 Inhibitors[Title/Abstract])) OR (PD-1 Inhibitor[Title/Abstract])) OR (Inhibitor, PD-1[Title/Abstract])) OR (PD 1 Inhibitor[Title/Abstract])) OR (Immune Checkpoint Blockade[Title/Abstract])) OR (Checkpoint Blockade, Immune[Title/Abstract])) OR (Immune Checkpoint Inhibition[Title/Abstract])) OR (Checkpoint Inhibition, Immune[Title/Abstract])) OR (PD-L1 Inhibitors[Title/Abstract])) OR (PD L1 Inhibitors[Title/Abstract])) OR (Programmed Death-Ligand 1 Inhibitors[Title/Abstract])) OR (Programmed Death Ligand 1 Inhibitors[Title/Abstract])) OR (PD-L1 Inhibitor[Title/Abstract])) OR (PD L1 Inhibitor[Title/Abstract])) OR (PD-1-PD-L1 Blockade[Title/Abstract])) OR (Blockade, PD-1-PD-L1[Title/Abstract])) OR (PD 1 PD L1 Blockade[Title/Abstract]) Results:40357  #9:Query: #2 AND (#4 OR #6) AND #8 Results:272  #10:Query:#2 AND (#4 OR #6) AND #8 Results:10 |
| **Web of Science** | #1:Search Query:TS=("Carcinoma, Non-Small-Cell Lung" OR "Non-Small-Cell Lung Carcinoma" OR "Lung Carcinomas, Non-Small-Cell" OR "Carcinoma, Non Small Cell Lung" OR "Carcinoma, Non-Small Cell Lung" OR "Non-Small-Cell Lung Carcinomas" OR "Lung Carcinoma, Non-Small-Cell" OR "Non Small Cell Lung Carcinoma" OR "Non-Small Cell Lung Cancer" OR "Carcinomas, Non-Small-Cell Lung" OR "Nonsmall Cell Lung Cancer" OR "Non-Small Cell Lung Carcinoma") Results:112099  #2:Search Query:TS=("Neoadjuvant Therapy Systemic" OR "Therapy, Neoadjuvant Systemic" OR "Neoadjuvant Systemic Therapies" OR "Neoadjuvant Radiation Treatment" OR "Neoadjuvant Radiation" OR "Neoadjuvant Radiation Therapies" OR "Neoadjuvant Radiation Therapy" OR "Neoadjuvant Radiations" OR "Therapy, Neoadjuvant Radiation" OR "Neoadjuvant Radiotherapy" OR "Treatment, Neoadjuvant Radiation" OR "Radiation Therapy, Neoadjuvant" OR "Radiation, Neoadjuvant" OR "Radiotherapy, Neoadjuvant" OR "Neoadjuvant Radiation Treatments" OR "Radiation Treatment, Neoadjuvant" OR "Neoadjuvant Radiotherapies" OR "Neoadjuvant Chemoradiation Treatments" OR "Therapy, Neoadjuvant Chemoradiation" OR "Chemoradiation Therapy, Neoadjuvant" OR "Neoadjuvant Chemoradiations" OR "Treatment, Neoadjuvant Chemoradiation" OR "Neoadjuvant Chemoradiation Therapy" OR "Chemoradiation Treatment, Neoadjuvant" OR "Neoadjuvant Chemoradiation Treatment" OR "Neoadjuvant Chemoradiation Therapies" OR "Neoadjuvant Chemoradiotherapy" OR "Neoadjuvant Chemoradiation" OR "Neoadjuvant Chemoradiotherapies" OR "Chemoradiotherapy, Neoadjuvant" OR "Chemoradiation, Neoadjuvant" OR "Neoadjuvant Chemotherapy Treatment" OR "Treatment, Neoadjuvant Chemotherapy" OR "Neoadjuvant Chemotherapy" OR "Chemotherapy, Neoadjuvant" OR "Neoadjuvant Chemotherapies" OR "Chemotherapy Treatment, Neoadjuvant" OR "Neoadjuvant Chemotherapy Treatments" OR "Neoadjuvant Treatments" OR "Neoadjuvant Therapies" OR "Treatment, Neoadjuvant" OR "Neoadjuvant Treatment" OR "Therapy, Neoadjuvant" OR "Chemotherapy, Adjuvant" OR "Drug Therapy, Adjuvant" OR "Adjuvant Chemotherapy" OR "Adjuvant Drug Therapy") Results:84172  #3:Search Query:TS=("Immune Checkpoint Inhibitors" OR "Cytotoxic T-Lymphocyte-Associated Protein 4 Inhibitor" OR "Cytotoxic T Lymphocyte Associated Protein 4 Inhibitors" OR "CTLA 4 Inhibitors" OR "CTLA-4 Inhibitor" OR "CTLA 4 Inhibitor" OR "Cytotoxic T-Lymphocyte-Associated Protein 4 Inhibitors" OR "CTLA-4 Inhibitors" OR "Cytotoxic T Lymphocyte Associated Protein 4 Inhibitor" OR "Programmed Cell Death Protein 1 Inhibitors" OR "PD-1 Inhibitor" OR "Inhibitor, PD-1" OR "PD 1 Inhibitors" OR "PD-1 Inhibitors" OR "Programmed Cell Death Protein 1 Inhibitor" OR "PD 1 Inhibitor" OR "Checkpoint Inhibitors, Immune" OR "Immune Checkpoint Inhibitor" OR "Checkpoint Inhibitor, Immune" OR "Immune Checkpoint Blockers" OR "Checkpoint Blockers, Immune" OR "PD 1 PD L1 Blockade" OR "Blockade, PD-1-PD-L1" OR "PD-1-PD-L1 Blockade" OR "Immune Checkpoint Blockade" OR "Checkpoint Blockade, Immune" OR "Immune Checkpoint Inhibition" OR "Checkpoint Inhibition, Immune" OR "PD-L1 Inhibitors" OR "PD L1 Inhibitors" OR "Programmed Death-Ligand 1 Inhibitors" OR "PD-L1 Inhibitor" OR "Programmed Death Ligand 1 Inhibitors" OR "PD L1 Inhibitor") Results:58022  #4:Search Query: TS=(random* OR randomi* OR randomly OR placebo* OR sham OR trial OR "clinical trial" OR "controlled clinical trial" OR "double blind" OR "single blind" OR "triple blind" OR crossover OR "cross over" OR "parallel group" OR cluster random* OR RCT) Results:3290868  #5:Search Query:#1 AND #2 AND #3 AND #4 Results:158 |
| **Cochrane** | ID Search Hits  #1 MeSH descriptor: [Carcinoma, Non-Small-Cell Lung] explode all trees 6961  #2 "Non-Small-Cell Lung Carcinoma" or "Lung Carcinomas, Non-Small-Cell" or "Carcinoma, Non Small Cell Lung" or "Carcinoma, Non-Small Cell Lung" or "Non-Small-Cell Lung Carcinomas" or "Lung Carcinoma, Non-Small-Cell" or "Non Small Cell Lung Carcinoma" or "Non-Small Cell Lung Cancer" or "Carcinomas, Non-Small-Cell Lung" or "Nonsmall Cell Lung Cancer" or "Non-Small Cell Lung Carcinoma" 18255  #3 #1 OR #2 18255  #4 MeSH descriptor: [Neoadjuvant Therapy] explode all trees 2903  #5 "Systemic Treatment, Neoadjuvant" OR "Neoadjuvant Systemic Therapy" OR "Neoadjuvant Systemic Treatment" OR "Systemic Therapy, Neoadjuvant" OR "Neoadjuvant Systemic Treatments" OR "Treatment, Neoadjuvant Systemic" OR "Therapy, Neoadjuvant Systemic" OR "Neoadjuvant Systemic Therapies" OR "Neoadjuvant Radiation Treatment" OR "Neoadjuvant Radiation" OR "Neoadjuvant Radiation Therapies" OR "Neoadjuvant Radiation Therapy" OR "Neoadjuvant Radiations" OR "Therapy, Neoadjuvant Radiation" OR "Neoadjuvant Radiotherapy" OR "Treatment, Neoadjuvant Radiation" OR "Radiation Therapy, Neoadjuvant" OR "Radiation, Neoadjuvant" OR "Radiotherapy, Neoadjuvant" OR "Neoadjuvant Radiation Treatments" OR "Radiation Treatment, Neoadjuvant" OR "Neoadjuvant Radiotherapies" OR "Neoadjuvant Chemoradiation Treatments" OR "Therapy, Neoadjuvant Chemoradiation" OR "Chemoradiation Therapy, Neoadjuvant" OR "Neoadjuvant Chemoradiations" OR "Treatment, Neoadjuvant Chemoradiation" OR "Neoadjuvant Chemoradiation Therapy" OR "Chemoradiation Treatment, Neoadjuvant" OR "Neoadjuvant Chemoradiation Treatment" OR "Neoadjuvant Chemoradiation Therapies" OR "Neoadjuvant Chemoradiotherapy" OR "Neoadjuvant Chemoradiation" OR "Neoadjuvant Chemoradiotherapies" OR "Chemoradiotherapy, Neoadjuvant" OR "Chemoradiation, Neoadjuvant" OR "Neoadjuvant Chemotherapy Treatment" OR "Treatment, Neoadjuvant Chemotherapy" OR "Neoadjuvant Chemotherapy" OR "Chemotherapy, Neoadjuvant" OR "Neoadjuvant Chemotherapies" OR "Chemotherapy Treatment, Neoadjuvant" OR "Neoadjuvant Chemotherapy Treatments" OR "Neoadjuvant Treatments" OR "Neoadjuvant Therapies" OR "Treatment, Neoadjuvant" OR "Neoadjuvant Treatment" OR "Therapy, Neoadjuvant" 9011  #6 MeSH descriptor: [Chemotherapy, Adjuvant] explode all trees 6131  #7 "Drug Therapy, Adjuvant" OR "Adjuvant Chemotherapy" OR "Adjuvant Drug Therapy" 11654  #8 #4 OR #5 OR #6 OR #7 22484  #9 MeSH descriptor: [Immune Checkpoint Inhibitors] explode all trees 437  #10 "Cytotoxic T-Lymphocyte-Associated Protein 4 Inhibitor" OR "Cytotoxic T Lymphocyte Associated Protein 4 Inhibitors" OR "CTLA 4 Inhibitors" OR "CTLA-4 Inhibitor" OR "CTLA 4 Inhibitor" OR "Cytotoxic T-Lymphocyte-Associated Protein 4 Inhibitors" OR "CTLA-4 Inhibitors" OR "Cytotoxic T Lymphocyte Associated Protein 4 Inhibitor" OR "Programmed Cell Death Protein 1 Inhibitors" OR "PD-1 Inhibitor" OR "Inhibitor, PD-1" OR "PD 1 Inhibitors" OR "PD-1 Inhibitors" OR "Programmed Cell Death Protein 1 Inhibitor" OR "PD 1 Inhibitor" OR "Checkpoint Inhibitors, Immune" OR "Immune Checkpoint Inhibitor" OR "Checkpoint Inhibitor, Immune" OR "Immune Checkpoint Blockers" OR "Checkpoint Blockers, Immune" OR "PD 1 PD L1 Blockade" OR "Blockade, PD-1-PD-L1" OR "PD-1-PD-L1 Blockade" OR "Immune Checkpoint Blockade" OR "Checkpoint Blockade, Immune" OR "Immune Checkpoint Inhibition" OR "Checkpoint Inhibition, Immune" OR "PD-L1 Inhibitors" OR "PD L1 Inhibitors" OR "Programmed Death-Ligand 1 Inhibitors" OR "PD-L1 Inhibitor" OR "Programmed Death Ligand 1 Inhibitors" OR "PD L1 Inhibitor" 2583  #11 #9 OR #10 2821  #12 "random" OR "randomize" OR "randomized" OR "randomly" OR "placebo" OR "sham" OR "trial" OR "clinical trial" OR "controlled clinical trial" OR "double blind" OR "single blind" OR "triple blind" OR "crossover" OR "cross over" OR "parallel group" OR "cluster random" OR "RCT" 1986623  #13 #3 AND #8 AND #11 AND #12 61 |
| **Embase** | #1:Query: 'non small cell lung cancer'/exp OR 'bronchial non small cell cancer' OR 'bronchial non small cell carcinoma' OR 'carcinoma, non-small-cell lung' OR 'lung cancer, non small cell' OR 'lung non small cell cancer' OR 'lung non small cell carcinoma' OR 'non oat cell lung cancer' OR 'non small cell bronchial cancer' OR 'non small cell cancer, lung' OR 'non small cell lung carcinoma' OR 'non small cell pulmonary cancer' OR 'non small cell pulmonary carcinoma' OR 'non squamous nsclc' OR 'non-oat cell lung cancer' OR 'non-small-cell lung carcinoma' OR 'nonsmall cell carcinoma of the lung' OR 'nonsmall cell lung cancer' OR 'nonsmall cell lung carcinoma' OR 'pulmonary non small cell cancer' OR 'pulmonary non small cell carcinoma' OR 'non small cell lung cancer' Results:54  #2:Query: 'neoadjuvant therapy'/exp OR 'neo-adjuvant drug therapy' OR 'neo-adjuvant drug treatment' OR 'neo-adjuvant medication' OR 'neo-adjuvant therapy' OR 'neo-adjuvant treatment' OR 'neoadjuvant drug therapy' OR 'neoadjuvant drug treatment' OR 'neoadjuvant medication' OR 'neoadjuvant treatment' OR 'neoadjuvant therapy' Results:97949  #3:Query: 'adjuvant chemotherapy'/exp OR 'chemotherapy, adjuvant' OR 'adjuvant chemotherapy' Results:112727  #4:Query: #2 OR #3 Results:193678  #5:Query: 'immune checkpoint inhibitor'/exp OR 'immune checkpoint blocker' OR 'immune checkpoint inhibitors' OR 'immune checkpoint inhibitor' Results:79555  #6:Query: #1 AND #4 AND #5 Results:951  #7:Query: #6 AND 'randomized controlled trial'/de Results:54 |

| Table S3. Characteristics of Included Randomized Controlled Trials: PD-L1 Expression-Stratified Event-Free Survival （EFS）Hazard Ratios | | | | |
| --- | --- | --- | --- | --- |
|  | **EFS HR** | | | |
| **First Author** | **PD-L1＜1%** | **PD-L1≥1%** | **PD-L1 1-49%** | **PD-L1≥50%** |
| Forde PM | 0.85(0.54-1.32) | 0.41(0.24-0.70) | 0.58(0.30-1.12) | 0.24(0.10-0.61) |
| Awad MM | 1.57(0.89-2.75) | 0.47(0.26-0.85) | 0.59(0.29-1.22) | / |
| Wakelee H | 0.77(0.55-1.07) | 0.47(0.36-0.63) | 0.51(0.34-0.75) | 0.42(0.28-0.65) |
| Spice JD | 0.75(0.56-1.01) | 0.51 (0.39-0.66) | 0.52 (0.36-0.73) | 0.48 (0.33-0.71) |
| Heynach JV | 0.76(0.49-1.17) | / | 0.70(0.46-1.05) | 0.60(0.35-1.01) |
| Lu S | 0.65(0.33-1.23) | / | 0.31 (0.17-0.54) | 0.31 (0.15-0.60) |
| Yue DS | 0.80(0.47-1.38) | 0.50(0.32-0.78) | 0.34(0.17-0.66) | 0.71(0.38-1.34) |
| Provencio M | / | / | / | / |
| Lei J | / | / | / | / |
| Cascone T | 0.73(0.47–1.15) | 0.52(0.35–0.78) | 0.76(0.46–1.25) | 0.26(0.12–0.55) |

| Table S4. Characteristics of Included Randomized Controlled Trials: PD-L1 Expression-Stratified Overall Survival (OS) Hazard Ratios | | | | |
| --- | --- | --- | --- | --- |
|  | **OS HR** | | | |
| **First Author** | **PD-L1**＜1% | PD-L1≥1% | **PD-L1 1-49%** | **PD-L1**≥50% |
| Forde PM | / | / | / | / |
| Awad MM | 1.32(0.67-2.59) | 0.47(0.25-0.88) | 0.59(0.29-1.21) | / |
| Wakelee H | / | / | / | / |
| Spice JD | 0.91(0.63-1.32) | 0.62(0.45-0.87) | 0.69(0.44-1.07) | 0.55(0.33-0.92) |
| Heynach JV | / | / | / | / |
| Lu S | / | / | / | / |
| Yue DS | / | / | / | / |
| Provencio M | 0.17(0.05-0.57) | 1.31(0.27-6.41) | / | / |
| Lei J | / | / | / | / |
| Cascone T | / | / | / | / |

| Table S5. Characteristics of Included Randomized Controlled Trials: PD-L1 Expression-Stratified Pathological Complete Response (pCR) Data | | | | | | | | |
| --- | --- | --- | --- | --- | --- | --- | --- | --- |
|  | **pCR RR** | | | | | | | |
| **First Author** | **PD-L1＜1%** | | **PD-L1≥1%** | | **PD-L1 1-49%** | | **PD-L1≥50%** | |
| Forde PM | 26/155 | 4/155 | 58/178 | 4/178 | 23/98 | 0/98 | 36/80 | 4/80 |
| Awad MM | 15/92 | 4/92 | 30/118 | 6/118 | 10/77 | 0/77 | 18/41 | 7/41 |
| Wakelee H | / | / | / | / | / | / | / | / |
| Spice JD | / | / | / | / | / | / | / | / |
| Heynach JV | 22/247 | 8/247 | / | / | 45/277 | 14/277 | 59/216 | 10/216 |
| Lu S | / | / | / | / | / | / | / | / |
| Yue DS | 33/89 | 7/84 | 57/130 | 6/131 | / | / | / | / |
| Provencio M | 1/3 | 0/3 | 9/17 | 1/17 | / | / | / | / |
| Lei J | 9/15 | 2/15 | 15/27 | 5/27 | / | / | / | / |
| Cascone T | 24/186 | 8/186 | 90/256 | 12/256 | 42/159 | 6/159 | 46/97 | 56/97 |

| Table S6.Characteristics of Included Randomized Controlled Trials: PD-L1 Expression-Stratified Major Pathological Response (MPR) Data | | | | | | | | |
| --- | --- | --- | --- | --- | --- | --- | --- | --- |
|  | **MPR** RR | | | | | | | |
| **First Author** | **PD-L1＜**1% | | PD-L1**≥**1% | | **PD-L1 1-49%** | | **PD-L1≥**50% | |
| Forde PM | 46/155 | 22/155 | 80/178 | 10/178 | 40/98 | 4/98 | 40/80 | 6/80 |
| Awad MM | / | / | / | / | / | / | / | / |
| Wakelee H | / | / | / | / | / | / | / | / |
| Spice JD | / | / | / | / | / | / | / | / |
| Heynach JV | / | / | / | / | / | / | / | / |
| Lu S | / | / | / | / | / | / | / | / |
| Yue DS | 43/89 | 14/84 | 81/130 | 19/131 | / | / | / | / |
| Provencio M | / | / | / | / | / | / | / | / |
| Lei J | 13/15 | 46/188 | 25/27 | 7/27 | / | / | / | / |
| Cascone T | 40/186 | 18/186 | 116/256 | 34/256 | 55/159 | 21/159 | 62/97 | 13/97 |

| Table S7. SUCRA-Based Ranking of Treatment Regimens for Efficacy Endpoints in the Bayesian Network Meta-Analysis | | | | |
| --- | --- | --- | --- | --- |
| **PD-L1 Expression Level** | **Endpoints** | **Drug** | **SUCRA（%）** | **Rank** |
| ＜1% | EFS | PIC | 96.3 | 1 |
|  |  | CT | 33.7 | 2 |
|  |  | NIC | 20 | 3 |
| ≥1% | EFS | NIC | 85.7 | 1 |
|  |  | PIC | 63.9 | 2 |
|  |  | CT | 0.4 | 3 |
| 1-49% | EFS | PIC | 80.9 | 1 |
|  |  | NIC | 65.8 | 2 |
|  |  | CT | 3.3 | 3 |
| ≥50% | EFS | NIC | 91.4 | 1 |
|  |  | PIC | 57.4 | 2 |
|  |  | CT | 1.1 | 3 |
| ＜1% | OS | PIC | 73.9 | 1 |
|  |  | CT | 56.4 | 2 |
|  |  | NIC | 19.7 | 3 |
| ≥1% | OS | NIC | 84.4 | 1 |
|  |  | PIC | 65.1 | 2 |
|  |  | CT | 0.5 | 3 |

| Table S8. SUCRA-Based Ranking of Treatment Regimens for Efficacy Endpoints in the Bayesian Network Meta-Analysis | | | | |
| --- | --- | --- | --- | --- |
| **PD-L1 Expression Level** | **Endpoints** | **Drug** | **SUCRA（%）** | **Rank** |
| ＜1% | pCR | Camrelizumab_CT | 83.1 | 1 |
|  |  | Tislelizumab_CT | 71.4 | 2 |
|  |  | Nivolumab_CT | 58.4 | 3 |
|  |  | Nivolumab_Ipilimumab | 51.2 | 4 |
|  |  | Durvalumab_CT | 35.6 | 5 |
|  |  | CT | 0.3 | 6 |
| ≥1% | pCR | Tislelizumab_CT | 85.8 | 1 |
|  |  | Nivolumab_CT | 80.3 | 2 |
|  |  | Nivolumab_Ipilimumab | 44.9 | 3 |
|  |  | Camrelizumab_CT | 38.9 | 4 |
|  |  | CT | 0 | 5 |
| 1-49% | pCR | Nivolumab_CT | 87.2 | 1 |
|  |  | Nivolumab_Ipilimumab | 71.7 | 2 |
|  |  | Durvalumab_CT | 40.9 | 3 |
|  |  | CT | 0.1 | 4 |
| ≥50% | pCR | Durvalumab_CT | 97.5 | 1 |
|  |  | Nivolumab_Ipilimumab | 67.2 | 2 |
|  |  | Nivolumab_CT | 34.0 | 3 |
|  |  | CT | 1.3 | 4 |
| ＜1% | MPR | Camrelizumab_CT | 79.2 | 1 |
|  |  | Tislelizumab_CT | 78.0 | 2 |
|  |  | Nivolumab_CT | 42.7 | 3 |
|  |  | CT | 0.1 | 4 |
| ≥1% | MPR | Camrelizumab_CT | 84.7 | 1 |
|  |  | Tislelizumab_CT | 63.7 | 2 |
|  |  | Nivolumab_CT | 51.6 | 3 |
|  |  | CT | 0 | 4 |

| **Table S9.**Meta-Analysis Outcomes of EFS, OS, pCR, and MPR by PD-L1 Expression Status | | | | | | |
| --- | --- | --- | --- | --- | --- | --- |
| **Outcome Measure** | **PD-L1 Expression Level** | **Model Type** | **Dbar** | **pD** | **DIC** | **I^2^** |
| EFS | PD-L1＜1% | Consistency | 5.197867 | 3.383928 | 8.581796 | 0% |
|  |  | Inconsistency | 5.210065 | 3.379693 | 8.589758 | 0% |
|  | PD-L1≥1% | Consistency | 3.032952 | 2.964278 | 5.99723 | 0% |
|  |  | Inconsistency | 3.021239 | 2.949033 | 5.970272 | 0% |
|  | PD-L1 1-49% | Consistency | 7.453904 | 5.081071 | 12.534976 | 20% |
|  |  | Inconsistency | 7.423575 | 5.105807 | 12.529382 | 19% |
|  | PD-L1≥50% | Consistency | 6.404471 | 4.437374 | 10.841845 | 22% |
|  |  | Inconsistency | 6.420768 | 4.437164 | 10.857932 | 22% |
| OS | PD-L1＜1% | Consistency | 2.184267 | 1.991416 | 4.175684 | 8% |
|  |  | Inconsistency | 2.212763 | 2.043303 | 4.256067 | 10% |
|  | PD-L1≥1% | Consistency | 6.046257 | 1.999205 | 8.045462 | 67% |
|  |  | Inconsistency | 3.336925 | 2.966939 | 6.303864 | 40% |
| pCR | PD-L1＜1% | Consistency | 12.63743 | 10.49091 | 23.12834 | 13% |
|  |  | Inconsistency | 12.13291 | 11.13464 | 23.26755 | 9% |
|  | PD-L1≥1% | Consistency | 11.57289 | 9.58527 | 21.15816 | 5% |
|  |  | Inconsistency | 11.61408 | 10.45749 | 22.07157 | 5% |
|  | PD-L1 1-49% | Consistency | 8.829709 | 6.272861 | 15.102570 | 21% |
|  |  | Inconsistency | 8.418413 | 6.729171 | 15.147584 | 17% |
|  | PD-L1≥50% | Consistency | 12.794109 | 6.690825 | 19.204465 | 45.2% |
|  |  | Inconsistency | 8.229699 | 7.580836 | 15.810536 | 15% |
| MPR | PD-L1＜1% | Consistency | 7.019834 | 6.689036 | 13.708870 | 0.3% |
|  |  | Inconsistency | 7.542300 | 7.207498 | 14.749798 | 7% |
|  | PD-L1≥1% | Consistency | 12.874411 | 6.680767 | 19.555177 | 46% |
|  |  | Inconsistency | 8.310001 | 7.670778 | 15.980779 | 16% |

| Table S10 Distribution of evidence quality grades for each clinical endpoint across different PD-L1 expression subgroups | | | | | |
| --- | --- | --- | --- | --- | --- |
| **Endpoints** | **PD-L1 Expression Level** | **High*** | **Mid†** | **Low‡** | **very low§** |
| EFS | <1% | 0 | 3 | 0 | 0 |
|  | ≥1% | 0 | 2 | 1 | 0 |
|  | 1-49% | 0 | 1 | 2 | 0 |
|  | ≥50% | 0 | 3 | 0 | 0 |
| OS | <1% | 1 | 2 | 0 | 0 |
|  | ≥1% | 1 | 2 | 0 | 0 |
| pCR | <1% | 2 | 9 | 3 | 1 |
|  | ≥1% | 1 | 4 | 5 | 0 |
|  | 1-49% | 2 | 1 | 3 | 0 |
|  | ≥50% | 0 | 0 | 3 | 3 |
| MPR | <1% | 1 | 3 | 2 | 0 |
|  | ≥1% | 0 | 1 | 3 | 2 |

*=high certainty of evidence; †=moderate certainty of evidence; ‡=low certainty of evidence; §=very low certainty of evidence

| Supplementary Table S11. Detailed Treatment Regimens of the Included Randomized Controlled Trials | | | | | | | | |
| --- | --- | --- | --- | --- | --- | --- | --- | --- |
| **First author** | **Source** | **Randomization** | **Median Age/y** | **Male/Female** | **Ethnicity (%)** | **Intervention Arm** | **Control Arm** | **AJCC Staging Version**  **(Edition)** |
| Forde PM | N Engl J Med | 1:1 | 64/65 | 255/103 | North America(25.4)  Europe(18.4)  Asian(49.4)  Other(6.7) | Nivolumab 360 mg Q3W Day 1+ chemotherapy Q3W Day 1 (for 3 cycles total) | Chemotherapy Q3W Day 1 (for 3 cycles total) | 7th |
| Awad MM | JCO | 1:1 | 64/65 | 143/78 | White(58.4)  Black or African American(3.2)  Asian(34.8)  Other(3.6) | Nivolumab 3mg/kg Q2W Day 1F(for 3 cycles)+ipilimumab 1 mg/kg Day 1(administered only in Cycle 1) | platinum-doublet chemotherapy Q3W Day 1 (for 3 cycles) | 7th |
| Wakelee H | N Engl J Med | 1:1 | 63/64 | 563/234 | American Indian or Alaska Native(0.1)  Asian(31.2)  Black(2.0)  Multiple(1.6)  White(61.4)  Missing data(3.6) | Neoadjuvant therapy(Pembrolizumab 200 mg Q3W Day 1 + cisplatin-based chemotherapy Q3W Day 1for 4 cycles total)+Surgery+adjuvant therapy(Pembrolizumab 200 mg Q3W Day 1for up to 13 cycles) | Neoadjuvant therapy (Placebo Q3W Day 1 + cisplatin-based chemotherapy Q3W Day 1 (for 4 cycles total))+Surgery+adjuvant therapy(Placebo Q3W Day 1 (for up to 13 cycles)) | 8th |
| Spicer JD | Lancet | 1:1 | 63/64 | 563/234 | American Indian or Alaska Native(0.1)  Asian(31.2)  Black(2.0)  Multiple(1.6)  White(61.4)  Missing data(3.6) | neoadjuvant therapy(Pembrolizumab 200 mg Q3W Day 1 +  cisplatin-based chemotherapy Q3W Day 1for 4 cycles total)+Surgery + adjuvant therapy(Pembrolizumab 200 mg Q3W Day 1for up to 13 cycles) | Neoadjuvant therapy (Placebo Q3W Day 1 + cisplatin-based chemotherapy Q3W Day 1 (for 4 cycles total) )+Surgery+adjuvant therapy(Placebo Q3W Day 1 (for up to 13 cycles)) | 8th |
| Heymach JV | N Engl J Med | 1:1 | 65/65 | 530/210 | Asian(41.5)  White(53.7)  Other(4.9) | Neoadjuvant therapy (Durvalumab 1500 mg Q3W Day 1 + platinum-based chemotherapy Q3W Day 1for 4 cycles total)+Surgery+Adjuvant therapy (Durvalumab 1500 mg Q4W Day 1for 12 cycles total) | Neoadjuvant therapy (Placebo Q3W Day 1 + platinum-based chemotherapy Q3W Day 1 (for 4 cycles total))+Surgery+Adjuvant therapy (Placebo Q4W Day 1 (for 12 cycles total)) | 8th |
| Lu S | JAMA | 1:1 | 62/61 | 370/34 | Asian(100) | Neoadjuvant (Toripalimab 240 mg Q3W Day 1 + platinum-based chemotherapy Q3W Day 1for 3 cycles total)+ Surgery + Adjuvant (Toripalimab 240 mg Q3W Day 1 up to 13 cycles(combined with chemotherapy)) | Neoadjuvant (Placebo Q3W Day 1 + platinum-based chemotherapy Q3W Day 1 (for 3 cycles total))+Surgery+ Adjuvant (Placebo Q3W Day 1 (for 1 cycle, combined with chemotherapy))l | 8th |
| Yue DS | Lancet | 1:1 | 62/63 | 410/43 | Asian(100.0) | Neoadjuvant (Tislelizumab 200 mg Q3W Day 1(for 3-4 cycles) + platinum-based doublet chemotherapy Q3W Day 1(for 3-4 cycles))+Surgery+Adjuvant (Tislelizumab 400 mg Q6W Day 1for up to 8 cycles) | Neoadjuvant (Placebo Q3W Day 1 (for 3-4 cycles) + platinum-based doublet chemotherapy Q3W Day 1 (for 3-4 cycles))+Surgery+Adjuvant (Placebo Q6W Day 1 (for up to 8 cycles)) | 8th |
| Provencio M | N Engl J Med | 2:1 | 65/63 | 52/34 | White (100.0) | Neoadjuvant (Nivolumab 360 mg Q3W Day 1 + paclitaxel 200 mg/m² Q3W Day 1 +  Carboplatin AUC=5 mg/mL/min Q3W Day 1for 3 cycles total)+Surgery+Adjuvant (Nivolumab 480 mg Q4W Day 1for 6 months (only for patients with R0 resection)) | Neoadjuvant (Paclitaxel 200 mg/m² Q3W Day 1 + carboplatin AUC=5 mg/mL/min Q3W Day 1 (for 3 cycles total)) + Surgery+Observation only | 8th |
| Lei J | JAMA Oncol | 1:1 | 61/61 | 74/14 | Asian(100.0) | Camrelizumab 200 mg Q3W Day 1(for 3 cycles) + chemotherapy Q3W Day 1(for 3 cycles) | Chemotherapy Q3W Day 1 (for 3 cycles total) | 8th |
| Cascone T | N Engl J Med | 1:1 | 66/66 | 327/134 | White (71.6)  Black (1.7)  Asian(25.2)  Other(1.5) | Neoadjuvant treatment (Nivolumab 360 mg Q3W Day 1 + platinum-doublet chemotherapy Q3W Day 1for 4 cycles total)+Surgery+adjuvant treatment (Nivolumab 480 mg Q3W Day 1 platinum-doublet chemotherapy Q3W Day 1for 1 year total) | Neoadjuvant treatment (Placebo Q3W Day 1 (for 4 cycles total) + platinum-doublet chemotherapy Q3W Day 1 (for 4 cycles total))+surgery+adjuvant treatment (Placebo Q4W Day 1 (for 1 year total)) | 8th |

Note: AUC, area under the concentration-time curve; NCT, National Clinical Trial identifier; Q2W, every 2 weeks; Q3W, every 3 weeks; Q4W, every 4 weeks; Q6W, every 6 weeks; R0, microscopically margin-negative resection

**Figure S1. Summary of results from assessment of studies by using Cochrane Risk of Bias Tool 2.0**

Studies were classified into one of three categories: low, high risk or having “some concerns”

**Figure S2 Convergence diagnostics of Bayesian network meta-analysis for EFS in NSCLC with PD-L1 expression < 1%**

**Figure S3. Bayesian network meta-analysis of EFS in NSCLC with PD-L1 expression ≥ 1%**

**Figure S4. Bayesian network meta-analysis of EFS in NSCLC with PD-L1 expression 1–49%**

**Figure S5. Bayesian network meta-analysis of EFS in NSCLC with PD-L1 expression ≥ 50%**

**Figure S6. Convergence diagnostics of Bayesian network meta-analysis for OS in NSCLC with PD-L1 expression < 1%**

**Figure S7. Convergence diagnostic plots of Bayesian network meta-analysis for OS in NSCLC with PD-L1 expression ≥ 1%**

**Figure S8. Convergence diagnostics of Bayesian network meta-analysis for pCR in NSCLC with PD-L1 expression < 1%**

**Figure S9. Convergence diagnostics of Bayesian network meta-analysis for pCR in NSCLC with PD-L1 expression ≥ 1%**

**Figure S10. Convergence diagnostics of Bayesian network meta-analysis for pCR in NSCLC with PD-L1 expression 1–49%**

**Figure S11. Convergence diagnostics of Bayesian network meta-analysis for pCR in NSCLC with PD-L1 expression ≥ 50%**

**Figure S12 Convergence diagnostics of Bayesian network meta-analysis for MPR in NSCLC with PD-L1 expression < 1%**

**Figure S13. Convergence diagnostics of Bayesian network meta-analysis for MPR in NSCLC with PD-L1 expression ≥ 1%**

**Figure S14. Trace plots and density plots of Bayesian network meta-analysis for EFS in NSCLC with PD-L1 expression < 1%**

**Figure S15. Trace plots and density plots of Bayesian network meta-analysis for EFS in NSCLC with PD-L1 expression ≥ 1%**

**Figure S16. Trace plots and density plots of Bayesian network meta-analysis for EFS in NSCLC with PD-L1 expression 1–49%**

**Figure S17. Trace plots and density plots of Bayesian network meta-analysis for EFS in NSCLC with PD-L1 expression ≥ 50%**

**Figure S18. Trace plots and density plots of Bayesian network meta-analysis for OS in NSCLC with PD-L1 expression < 1%**

**Figure S19. Trace plots and density plots of Bayesian network meta-analysis for OS in NSCLC with PD-L1 expression ≥ 1%**

**Figure S20. Trace plots and density plots of Bayesian network meta-analysis for pCR in NSCLC with PD-L1 expression < 1%**

**Figure S21. Trace plots and density plots of Bayesian network meta-analysis for pCR in NSCLC with PD-L1 expression ≥ 1%**

**Figure S22. Trace plots and density plots of Bayesian network meta-analysis for pCR in NSCLC with PD-L1 expression 1–49%**

**Figure S23. Trace plots and density plots of Bayesian network meta-analysis for pCR in NSCLC with PD-L1 expression ≥ 50%**

**Figure S24. Trace plots and density plots of Bayesian network meta-analysis for MPR in NSCLC with PD-L1 expression < 1%**

**Figure S25. Trace plots and density plots of Bayesian network meta-analysis for MPR in NSCLC with PD-L1 expression ≥ 1%**

**Figure S26. Funnel plot for publication bias assessment of pCR in non-small cell lung cancer patients with PD-L1 expression < 1%**

**Figure S27. Funnel plot for publication bias assessment of pCR in non-small cell lung cancer patients with PD-L1 expression ≥ 1%**

**Figure S28. Funnel plot for publication bias assessment of pCR in non-small cell lung cancer patients with PD-L1 expression 1~49%**

**Figure S29. Funnel plot for publication bias assessment of pCR in non-small cell lung cancer patients with PD-L1 expression ≥50%**

**Figure S30. Funnel plot for publication bias assessment of MPR in non-small cell lung cancer patients with PD-L1 expression ＜1%**

**Figure S31. Funnel plot for publication bias assessment of MPR in non-small cell lung cancer patients with PD-L1 expression≥1%**

**Figure S32. Network graphs of comparative interventions for pCR and MPR in patients with NSCLC**. Network structure depicting the pairwise comparisons of different interventions for pCR and MPR across different PD-L1 expression levels: (A) pCR, PD-L1 < 1%; (B) pCR, PD-L1 ≥ 1%; (C) pCR, PD-L1 1-49%; (D) pCR, PD-L1 ≥ 50%; (E) MPR, PD-L1 < 1%; (F) MPR, PD-L1 ≥ 1%.

Abbreviations: CT, chemotherapy; Camrelizumab+CT, camrelizumab plus chemotherapy; Durvalumab+CT, durvalumab plus chemotherapy; Nivolumab+CT, nivolumab plus chemotherapy; Nivolumab+Ipilimumab, nivolumab plus ipilimumab; Tislelizumab+CT, tislelizumab plus chemotherapy.
